# Isolation, phenotypic characterization and comparative genomic analysis of 2019SD1, a polyvalent enterobacteria phage

**DOI:** 10.1038/s41598-021-01419-8

**Published:** 2021-11-12

**Authors:** Prince Kumar, Mukesh K. Meghvansi, D. V. Kamboj

**Affiliations:** 1 Biotechnology Division, Defence Research & Development Establishment, Gwalior, Madhya Pradesh 474002 India; 2 Present Address: Regional Ayurveda Research Institute, Gwalior, Madhya Pradesh 474009 India; 3 Present Address: Bioprocess Technology Division, Defence Research & Development Establishment, Gwalior, Madhya Pradesh 474002 India; 4 Present Address: Defence Research Laboratory, Tezpur, Assam 784001 India

**Keywords:** Microbiology, Bacteriophages

## Abstract

*Shigella* has the remarkable capability to acquire antibiotic resistance rapidly thereby posing a significant public health challenge for the effective treatment of dysentery (Shigellosis). The phage therapy has been proven as an effective alternative strategy for controlling *Shigella* infections. In this study, we illustrate the isolation and detailed characterization of a polyvalent phage 2019SD1, which demonstrates lytic activity against *Shigella dysenteriae*, *Escherichia coli, Vibrio cholerae, Enterococcus saccharolyticus* and *Enterococcus faecium.* The newly isolated phage 2019SD1 shows adsorption time < 6 min, a latent period of 20 min and burst size of 151 PFU per bacterial cell. 2019SD1 exhibits considerable stability in a wide pH range and survives an hour at 50 °C. Under transmission electron microscope, 2019SD1 shows an icosahedral capsid (60 nm dia) and a 140 nm long tail. Further, detailed bioinformatic analyses of whole genome sequence data obtained through Oxford Nanopore platform revealed that 2019SD1 belongs to genus *Hanrivervirus* of subfamily *Tempevirinae* under the family *Drexlerviridae.* The concatenated protein phylogeny of 2019SD1 with the members of *Drexlerviridae* taking four genes (DNA Primase, ATP Dependent DNA Helicase, Large Terminase Protein, and Portal Protein) using the maximum parsimony method also suggested that 2019SD1 formed a distinct clade with the closest match of the taxa belonging to the genus *Hanrivervirus.* The genome analysis data indicate the occurrence of putative tail fiber proteins and DNA methylation mechanism. In addition, 2019SD1 has a well-established anti-host defence system as suggested through identification of putative anti-CRISPR and anti-restriction endonuclease systems thereby also indicating its biocontrol potential.

## Introduction

*Shigella* is a Gram-negative, nonmotile, rod-shaped facultative anaerobic and non-spore-forming bacterium belonging to *Enterobacteriaceae* family. This genus includes four species namely, *Shigella boydii, Shigella dysenteriae, Shigella flexneri* and *Shigella sonnei. Shigella* is the etiological agent of Shigellosis with clinical manifestations ranging from mild watery diarrhoea to severe dysentery and other systemic complications such as electrolyte imbalance and hemolytic uremic syndrome^1^. *S. dysenteriae,* known to produce Shiga toxin, more frequently occurs in developing countries whereas *S. sonnei* causes 77% of Shigellosis episodes in the developed world^2^. According to the Global Burden of Disease Study of 1990–2016, *Shigella* was second leading cause of diarrhoeal mortality in 2016 among all ages, leading to 2,12,438 deaths and about 13.2% of all diarrhoea deaths. Moreover, *Shigella* was responsible for 63,713 deaths among children aged < 5 years and was frequently associated with diarrhoea across all adult age groups, increasing in elderly people, with broad geographical distribution^3^. *Shigella* infection occurs mainly via the faecal-oral route, with food, fomites, water, insects as well as direct person to person contact with the infectious dose being as low as 100 bacterial cells^4^. Therefore, Shigellosis is considered as a major public-health problem particularly in the developing countries where a large population resides with often inadequate sanitary facilities and poor hygienic conditions.

*Shigella* has the capability to cause epidemic episodes of dysentery in large population as evident from many cases reported in Asian and African countries. In 1984, a major outbreak caused by *S. dysenteriae* type 1 occurred in West Bengal and Tripura states of India affecting 3,50,000 people with 3500 deaths^5^. In 2002, another outbreak of *S. dysenteriae* type 1 occurred in West Bengal and tea gardens of Siliguri area with the overall attack rate, death rate among those admitted to hospital, and the overall case-fatality reported to be as high as 25.6%, 6.0% and 0.9% respectively^6^. More recently, an investigation from Mumbai reported four cases of *Shigella* septicaemia, out of which three were caused by *S. dysenteriae* type 1 and one by S*. flexneri* with a mortality rate of 75%^7^. Similarly, various episodes of diarrhoea caused by *S. dysenteriae* type 1 have been reported in many African countries^8–11^.

For antibiotic treatment of Shigellosis, WHO guidelines provide for the use of fluoroquinolones (first-line), β-lactams (second-line) and cephalosporins (second-line). In addition, Azithromycin and Cefixime are suggested as alternatives with some precautionary measures depending upon the case history and severity^12^. However, *Shigella* has the quaint potentiality to acquire antibiotic resistance rapidly thereby posing a significant public health challenge. In India, antimicrobial resistance in the genus *Shigella* is more common as compared to that in other enteric bacteria^13^. In 2003, studies from various parts of India reported that the newly emerged strains of *S. dysenteriae* type 1 exhibited resistance to fluoroquinolones^14,15^. A study conducted in Andaman & Nicobar Islands of India revealed that *S. dysenteriae* was more resistant, followed by *S. flexneri* (14%), than the other *Shigella* species, particularly to the third-generation cephalosporins^16^. Worryingly, New Delhi metallo-beta-lactamase 1 (*ndm-1*)-positive *Shigella* species has also been recovered from water pools in streets or rivulets in Delhi, which has serious public health implications as *ndm-1* is known to confer resistance to carbapenems and many other β-lactam antibiotics leaving behind very limited treatment options to the patients^17^. The *ndm-1* gene can potentially be transferred horizontally to other bacteria thereby rendering the bacteria multidrug resistant (Superbugs). Furthermore, diverse resistance genes causing the emergence of antibiotic-resistant strains of *S. dysenteriae* have been reported from various parts of India (e.g. plasmid mediated gene *bla-oxa1* for β-lactam resistance^18^, plasmid mediated gene *aac *(*6′*)*-Ib-cr* conferring quinolene resistance^19^). Similar reports of antibiotic resistance in *S. dysenteriae* have been reported worldwide^20,21^.

The evolving antibiotic resistance profile of *Shigella* isolates and inescapable spread of antibiotic resistance genes among its various species pose significant challenges in terms of prescribing standard medications for the effective treatment of Shigellosis, and warrants exploration of alternative treatment strategies. phages are natural predator viruses of bacteria and are the most abundantly present organisms in the environment. They are a ubiquitous feature of prokaryotic existence^22^. The phage therapy has been established as an effective strategy for controlling bacterial infections. Successful use of phages in the treatment of *S. dysenteriae* infections in children was demonstrated as early as 1931 at Yale University School of Medicine^23^. However, in subsequent decades, the emergence of antibiotic therapy had predominated the phage research and therapy. Nevertheless, over the last decade, the research interest in the realm of phages for the treatment against difficult bacterial pathogens has reinvigorated owing to the emergence of multi-drug resistant bacteria and other challenges associated with the antibiotic usage. Among the phages, the most of the researches have focused on those infecting *E. coli* and *Salmonella* whereas relatively less attention has been paid to phages of *Shigella*, in spite of the fact that Shigellosis is a major global health concern causing millions of infections every year. Some of the phages reported against *Shigella* species include sf6^24^, SfIV^25^, SfI^26^, vB_SsoS-ISF002^27^ and vB_SsoS-ISF003^28^. More recently, a new phage, Sfin-1, showing potent lytic activity against multidrug-resistant isolates of *S. flexneri*, *S. dysenteriae* and *S. sonnei* has been described from India^29^. However, limited studies are available on detailed biological properties and whole genome analysis of lytic phages infecting *S. dysenteriae*^30^ and other bacteria from *Enterobacteriaceae* family. Here, we report isolation, elucidation of detailed biological properties and comparative whole genomic analysis of 2019SD1, a polyvalent phage showing strong lytic activity against *S. dysenteriae*, *Escherichia coli, Vibrio cholerae, Enterococcus saccharolyticus* and *E. faecium.* Further, we compare its marker gene sequences (DNA Primase, ATP Dependent DNA Helicase, Large Terminase Protein and Portal Protein) with other known phage sequences which would be useful not only in synthesizing required knowledge for developing phage-based therapy against the Shigellosis, but also to shed light on the various evolutionary aspects and the host-lytic spectrum of the phage.

## Materials and methods

### Bacterial strains used in the study

Bacterial strains mentioned in this study were obtained from from various sources (Table 1) and the cultures were maintained on Luria Bertani (LB) agar (1.5% agar w/v) (BD DIFCO, USA) employing standard procedures.

**Table 1:** Host range and efficiency of plating (EOP) of *Shigella* virus 2019SD1.

S. no.	Name of bacterial strains	Source	Phage lytic pattern^a^	Efficiency of plating
1	*Shigella dysenteriae* type I	^b^DRDE, Gwalior, India	+	1.0 ± 0.09
2	*E. coli* ATCC 25922	^b^DRDE, Gwalior, India	+	0.64 ± 0.1
3	*Salmonella typhi* 4736pg	^b^DRDE, Gwalior, India	−	–
4	*Enterobacter clobae *NAIMCC 1255	^c^NAIMCC Mau, Uttar Pradesh, India	−	–
5	*Enterococcus faecium *NAIMCC 1045	^c^NAIMCC Mau, Uttar Pradesh, India	−	–
6	*Vibrio cholerae*	^b^DRDE, Gwalior, India	+	0.43 ± 0.3
7	*Staphylococcus aureus *ATCC25923	DRDE, Gwalior, India	−	–
8	*Klebsiella pneumoniae* MO2 (NCBI Accession No. MN387789)	^b^DRDE, Gwalior, India	−	–
9	*Enterococcus saccharolyticus *NAIMCC 1332	^c^NAIMCC Mau, Uttar Pradesh, India	+	0.62 ± 0.4
10	*Citrobacter amalonaticus *NAIMCC 1364	^c^NAIMCC Mau, Uttar Pradesh, India	−	–
11	*Enterococcus faecium *NAIMCC 1456	^c^NAIMCC Mau, Uttar Pradesh, India	+	0.74 ± 0.5
12	*Citrobacter freundii *NAIMCC 1351	^c^NAIMCC Mau, Uttar Pradesh, India	−	–

^a^Results recorded as + Sensitive; − No infection.

^b^In-house collection from previous study.

^c^National Agriculturally Important Microbial Culture Collection (NAIMCC).

### Phage isolation

*Shigella v*irus 2019SD1 was isolated from the sewage water collected from Atrauli, Aligarh, Uttar Pradesh, India (28.03° N, 78.28° E). Solid impurities in the sewage water were removed by centrifugation at 4000×*g* for 10 min. Supernatant was filtered using 0.22 µm syringe filter (MILLIPEX GP, MILLIPORE) for removing bacterial debris. Phage was isolated using enrichment method as described by Twest and Kropinski^31^. In the process, 90 mL of filtrate was taken and mixed with 5 mL of exponentially growing *S. dysenteriae* type 1 culture and incubated in shaker incubator (100 rpm) for 24 h at 37 °C. After incubation, the mixture was centrifuged again at 4000×*g* for 10 min and filtered through 0.22 µm syringe filter. For subsequent propagation of phage, 0.5 mL of the host strain grown overnight in LB broth was taken and mixed with 4.5 mL of soft agar (0.6% agar w/v). This mixture was poured onto LB agar plate (1.5% agar w/v) and the plate was left undisturbed for 20 min for solidification. After solidification, 100 μL of phage suspension (10^8^ PFU mL^−1^) was spotted on the agar plate and left for 20 min for absorption. Plate was incubated overnight at 37 °C and observed for plaque. Thereafter, 5 mL of SM buffer was added into the plate, shaken for 5 min and the lysate was taken out from the plate with the help of pipette. This lysate was centrifuged at 8000*g* for 10 min. After centrifugation, supernatant was transferred into new tube and filtered using 0.22 µm filter and used for further experimental work.

### Imaging of phage

The newly isolated phage solution (10 μL; 10^11^ PFU mL^−1^) was deposited on a 300-mesh copper grid and stained with 2% Phosphotungstic acid (pH 4.5) for 30 s. After air-drying, the grid was observed under transmission electron microscope (JEOL JEM-1400PLUS) at an accelerated voltage of 80 kV, and the image of phage was captured.

### Phage adsorption and one-step growth curve analysis

Phages were added to the host bacterial suspension at an MOI: 0.1. The number of non-adsorbed phages (NAP) was estimated at one-minute interval and percentage was calculated. One-step growth experiment was carried out as described by Hyman and Abedon^32^ to determine the burst size and latent period. In brief, the phage suspension was mixed with the exponentially growing culture of *S. dysenteriae* type 1 at an MOI of 0.1 and allowed to adsorb for 5 min at 37 °C. Non-adsorbed phages present in the mixture were removed by centrifugation at 10,000×*g* for 2 min. The pellet was resuspended in 10 mL of LB broth and allowed to incubate at 37 °C for 100 min. Subsequently from this sample, 100 µL of aliquot was drawn at every 10 min interval and phage titer was determined using double agar plating assay^33^.

### Determination of Host lytic spectrum and efficacy of plating (EOP)

Host lysis spectrum and EOP against 11 bacterial species as listed in Table 1 were determined according to the protocol described earlier^34^. Briefly, 0.5 mL of the test bacterial strain grown overnight in LB broth was mixed with 4.5 mL of soft agar (0.6% agar; w/v) and poured onto LB agar plate (1.5% agar; w/v). Thereafter, 10 μL of phage suspension (10^10^ PFU mL^−1^) was spotted onto LB agar medium and incubated overnight at 37 °C. Experiment was performed in triplicate, and the EOP for the susceptible bacterial hosts was estimated using double agar plating assay as described earlier^33^.

### Preservation of phage

For the purpose of evaluation of phage viability at various temperatures, SM buffer (NaCl 100 mM, MgSO_4_·7H_2_O 8 mM, Tris–Cl 50 mM,) was prepared. Phages were preserved at 25 °C and 4 °C in SM buffer and at − 20 °C, − 80 °C in SM buffer with 15% (v/v) glycerol in multiple borosilicate glass vials (2 mL volume). Samples were drawn from the vials at 15 d interval and the phage titer was estimated using double agar plating assay^33^.

### Phage stability under abiotic conditions

The phage was challenged with abiotic stress conditions which included varying temperatures (35 °C, 40 °C, 45 °C and 50 °C), pH (5.0, 7.0, 9.0 and 11.0) and salinity levels (NaCl; 5 g L^−l^, 10 g L^−l^, 15 g L^−l^ and 20 g L^−l^). In the process, 10 mL of phage suspension (10^8^ PFU mL^−1^) was added to 90 mL of autoclaved SM buffer with varying pH and salt concentrations and incubated at 37 °C. For thermal stability testing, in the similar manner the phage suspension in SM buffer was incubated at varying temperatures. Samples were drawn from these experimental flasks at 30 min, 60 min, 120 min, 240 min and 360 min after incubation and, the phage titer was estimated employing double agar plating assay^33^.

### Host cell lysis test

Host Cell lysis test was carried out in autoclaved saline buffer (0.9% NaCl; pH 7.0). Further, in order to mimic the performance of phage to lyse the bacterial cells present in the wastewater, effluent of anaerobic Biodigester treating human excreta was collected locally. 10 mL of bacterial culture (10^9^ CFU mL^−1^) was added to 89 mL of autoclaved saline buffer/effluent in a conical flask (Borosilicate glass; 250 mL volume) which provided ultimately 10^8^ CFU mL^−1^ of bacteria in the suspension. 1 mL of phage suspension was added to it. Host cell lysis was determined at three different MOIs (1, 10 and 100) in the flasks incubated at 37 °C. CFU count was performed at 1 h intervals up to 6 h and at 24 h by drawing 100 µL of sample plating on LB agar plates employing drop-plate method^35^. Briefly, 100 µL of sample was serially diluted in 0.9% NaCl. 20 µL of each dilution was dropped carefully onto the surface of the LB agar plate. The natural spread and diffusion of the drop was allowed. Thereafter, the plates were incubated for 18 h at 37 °C. Subsequent to incubation, bacterial colonies were counted and CFU was calculated using the formula provided below:

$${\text{CFU}}\,{\text{ mL}}^{{ - {1}}} \, = \,{\text{Average}}\,{\text{ number}}\,{\text{ of}}\,{\text{ colonies}}\,{\text{ for}}\,{\text{ a}}\,{\text{ dilution}}\, \times \,{5}0\, \times \,{\text{dilution }}\,{\text{factor}}.$$

### Phage nucleic acid extraction

Nucleic acid of 2019SD1 was extracted according to the procedure suggested earlier^36^ with slight modifications. One millilitre of the phage suspension (10^9^ PFU mL^−1^) was incubated with DNase (2 mg mL^−1^) and RNase (5 mg mL^−1^) for 1 h at 37 °C. Phage lysis buffer (100 μL of 10% SDS, 50 μL of 0.5 M EDTA, and 10 μL of 10 mg mL^−1^ proteinase K; pH 7.0) was added to the phage suspension, mixed well, and incubated at 50 °C for 30 min. Thereafter, protein was precipitated from this suspension using 3.5 M ammonium acetate (@60 µL mL^−1^). After adding ammonium acetate, suspension was incubated on ice for 30 min and centrifuged at 13,000×*g* for 10 min. Thereafter, the supernatant was transferred to a fresh tube, wherein an equal volume of phenol:chloroform:-isoamyl alcohol (25:24:1) was added. The sample was subsequently centrifuged at 10,000×*g* for 10 min. This step was repeated once. The aqueous upper phase was then taken into a new tube and an equal volume of chloroform was added prior to centrifugation of sample at 10,000×*g* for 10 min. Next, the aqueous upper phase was again transferred to a new tube and an equal volume of isopropanol was added and the sample was allowed to precipitate at 20 °C for 2 h. In subsequent step, the precipitated nucleic acid was centrifuged at 12,000×*g* for 5 min at 4 °C and, the pelleted nucleic acid was washed with 70% (v/v) ethanol. The purified nucleic acid was visualized on a 0.9% (w/v) agarose gel. Whether the nucleic acid was DNA or RNA, was confirmed through DNAse I and RNAse A (FERMENTAS, USA) treatment separately as per the manufacturer’s instructions.

### DNA library preparation and oxford nanopore sequencing

End-repairing of DNA sample of 2019SD1 was performed using NEBNext Ultra II End Repair kit (NEW ENGLAND BIOLABS, MA, USA) and subsequent clean up with 1 × Ampure beads (BECKMANN COULTER, USA). Thereafter, native barcode ligation was carried out with NEB Blunt/TA ligase (NEW ENGLAND BIOLABS, MA, USA) using nbd103 (ONT) and cleaned with 1 × Ampure beads. Further, the barcode-ligated DNA sample was quantified using Qubit 4 (THERMOFISHER SCIENTIFIC, USA). In subsequent step, bam adapter ligation was performed for 15 min using NEBNext quick ligation module (NEW ENGLAND BIOLABS, MA, USA). Again, the library mix was cleaned up with the help of 0.4 × Ampure beads (BECKMANN COULTER, USA). Finally, the sequencing library was eluted in 15 µL of elution buffer and used for Nanopore sequencing. Gridion × 5 (OXFORD NANOPORE TECHNOLOGIES, OXFORD, UK) with spoton flow cell (r9.4) was used for sequencing in a 48 h sequencing protocol on Minknow 2.1 v18.05.5. In order to eliminate probable errors in long-read assemblies, nanopore raw reads (‘*fast5*’ format) were basecalled (‘*fastq5*’ format) and demultiplexed using Albacore v2.3.1. Further for sequence polishing, basecalled reads were error-corrected and assembled using “Canu” assembler v1.8.

### Whole genome analysis

Assembled phage genome was analysed using RAST (http://rast.nmpdr.org/rast.cgi) with customized RASTtk pipeline call features *glimmer3*, *prodigal* and *genemark*^37^ for predicting putative open reading frames (ORFs). Annotation was carried out using RAST annotation scheme by enabling the ‘annotate protein’ option in the program. Functional prediction of ORFs was then confirmed by BLASTp analysis with database of non-redundant (nr) protein sequences (https://blast.ncbi.nlm.nih.gov/Blast.cgi?PAGE=Protein)^38^. In addition, hypothetical putative ORFs of phage 2019SD1 were investigated for distinct homologs proteins in HHpred analysis^39^. Genomic map was visualized using CGview software (http://cgview.ca/)^40^. EasyFig genome visualizer was used for comparatively analyzing various modules^41^. Putative tRNA was identified using tRNAscan-SE 1.21 (http://lowelab.ucsc.edu/tRNAscan-SE/)^42^. Cleavage sites of phage were analyzed with reported restriction enzymes using REBASE program (http://rebase.neb.com/rebase/). Conserved domains were predicted using Pfam^43^ and NCBI Web CD-search tool^44^.

### Comparative genomic analysis based on nucleotide sequences

NCBI-BLASTn analysis of the whole genome sequence of 2019SD1 was carried out to know its percent identity with other known phages. This analysis provided 34 phages having an E-value of 0.0, which were further considered for comparative genomic analysis. The Genome-BLAST Distance Phylogeny (GBDP) method^45^ was used for pairwise comparisons of the whole genome nucleotide sequences under the default settings prescribed for the prokaryotic viruses^46^ in VICTOR program (https://ggdc.dsmz.de/). The phylogenomic GBDP trees were inferred using the formulas D0, D4 and D6 which provided average support of 2%, 21% and 6%, respectively. Accordingly, the tree with D4 formula providing maximum average support was recruited in analysis. In addition, a heatmap integrating the intergenomic similarity values with information regarding the genome lengths and the aligned genome fraction was prepared using VIRDIC program (https://viridic.icbm.de).

### Comparative analysis based on protein homologies

In order to determine the core sets of genes and to evaluate the protein homologies of phage 2019SD1 with other viruses, five phages exhibiting maximum similarity as suggested through an all-against-all fragment analysis approach using VIRIDIC server were selected, and their corresponding protein data were subjected to core gene analysis using CoreGenes v3.5 with default threshold setting of 75^47^.

According to the information available in the literature on the marker genes commonly prevalent in *Caudovirales* group^48^, four proteins namely, terminase large subunit, portal protein, ATP dependent helicase and DNA primase were chosen for this analysis using the Maximum Parsimony (MP) method. In the process, protein data were concatenated using Geneious Prime 2020.2.2 (Build 2020-07-23 08:02 Java Version 11.0.4 + 11 64 bit) and evolutionary analysis was carried out using the MP method in MEGA X^49^ using the Subtree-Pruning-Regrafting (SPR) algorithm^50^ with search level 1 in which the initial trees were obtained by the random addition of sequences (10 replicates). The bootstrap consensus tree inferred from 1000 replicates was taken to represent the evolutionary history of the taxa under analysis. Branches that corresponded to partitions reproducing in less than 50% bootstrap replicates were collapsed. All positions containing gaps as well as missing data were removed with a total of 1237 positions left in the final dataset.

### Statistical analysis

Empirical data were analyzed statistically using SPSS statistics v17.0 (SPSS INC., CHICAGO, IL, USA). The existence of significant differences among the different conditions tested for each tested parameter was assessed by one way ANOVA model followed by Tukey’s HSD Post hoc test. A value of *P *≤ 0.05 was considered statistically significant.

### Accession number

Complete genome sequence of 2019SD1 has been deposited in NCBI GenBank through Bankit under accession number MT360681. Additionally, *fastq* file in respect of raw sequence data is submitted to NCBI-SRA database under the identifier SRS7234126.

## Results and discussion

### Phage isolation, identification, adsorption, and one step curve

**Figure 1:**
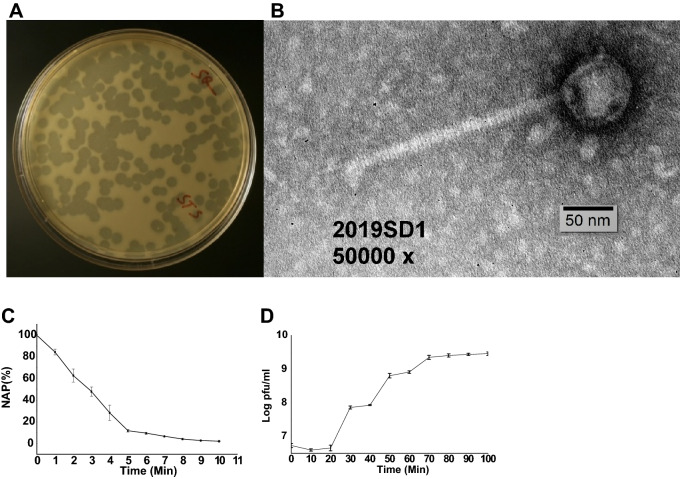
Plaque, TEM morphology, rate of adsorption and one-step growth curve of *Shigella virus* 2019SD1. (**A**) Plaques produced against *S. dysenteriae* type 1 using double agar plating assay, (**B**) Transmission electron micrograph (**C**) The rate of adsorption of phage to the *S. dysenteriae* type 1 host. Phages were added to the bacterial suspension at an MOI: 0.1. The percentage of non-adsorbed phages (NAP) was calculated at the indicated time points. The presented data are means of three independent experiments with error bars showing the standard error of mean (SEM). (**D**) One-step growth curve analysis. Plaque forming units (PFU) were recorded at different time interval post infection against host bacterium *S. dysenteriae* type 1. Bars represent SEM.

As the phages need specific hosts for their propagation, and *Shigella* spp. are primarily regarded as water-borne pathogens, in the present study wastewater sample was used for the phage isolation. The newly isolated lytic phage of *S. dysenteriae* was designated as *Shigella* virus 2019SD1 in *Drexlerviridae* family according to the recommendations provided by Adriaenssens and Brister^51^ and latest ICTV guidelines (https://talk.ictvonline.org/taxonomy/ accessed 6 January 2021). 2019SD1 formed clear plaques of ~ 2.5 mm diameter on a double-layered agar plate following 8 h incubation at 37 °C (Fig. 1A). The plaque size was nearly similar to that observed for other *Shigella* phages namely vB_SsoS_008 (2.5–2.7 mm)^52^, but markedly higher or lower than that recorded for pSf-1 (4.5 mm)^53^, JK16 (4 mm)^28^, vB-SdyS-ISF003 (3 mm)^50^ SsoS-ISF002 (0.5 to 3.0 mm)^27^, SH6 (2 mm)^54^, Sfin-1(1.5–2.0 mm)^29^, vB_SflS-ISF001 (0.5–2.0 mm)^30^. Analysis of TEM images of phage particles suggested that the 2019SD1 had an icosahedral capsid (60 ± 5 nm dia) and a 140 ± 6 nm long tail (Fig. 1B). The tail length was nearly similar to those observed for pSf-2 (136 ± 3 nm)^55^ but considerably varied from that recorded for SsoS-ISF002 (196 ± 14 nm)^27^, vB_SsoS_008 (171.2 ± 5 nm)^52^ vB-SdyS-ISF003 (160 ± 5 nm)^54^ and pSf-1 (103 ± 6 nm)^53^. The head diameter of 2019SD1 was also nearly similar to that observed for pSf-2 (57 ± 4 nm)^55^ and vB_SsoS_008 (59.2 ± 2 nm)^52^ but smaller than that of pSf-1 (73 ± 3 nm)^53^, vB-SdyS-ISF003 (70 ± 3 nm)^28^ and JK16 (64 ± 1)^54^.

Data on adsorption experiment indicated that > 80% of the phage particles adsorbed to the host cells within 5 min (Fig. 1C). One-step growth curve study revealed that 2019SD1 had a 20-min latent period and 50-min outbreak period (Fig. 1D). Accordingly, the maximum number of progenies released from one host bacterium was recorded as 151 PFU bacterial cell^−1^. Although the latent period was fairly similar to the one observed for vB_SflS-ISF001 (20 min)^30^, but was markedly higher or lower than that recorded for pSf-2 (30 min)^56^, SH6 phage (16 min)^55^ and Sfin-1 (15 min for *S. dysenteriae* and 10 min for *S. sonnei*)^29^. Interestingly, the burst size of 2019SD1 was greater than that of other reported *Shigella* phages of *Drexlerviridae* family like pSf-2 (16 PFU infected cell^−1^)^56^, SH6 phage (103 ± 16 PFU infected cell^−1^)^55^ and SsoS-ISF002 (76 ± 9 PFU infected cell^−1^)^27^.

### Host cell lysis activity

**Figure 2:**
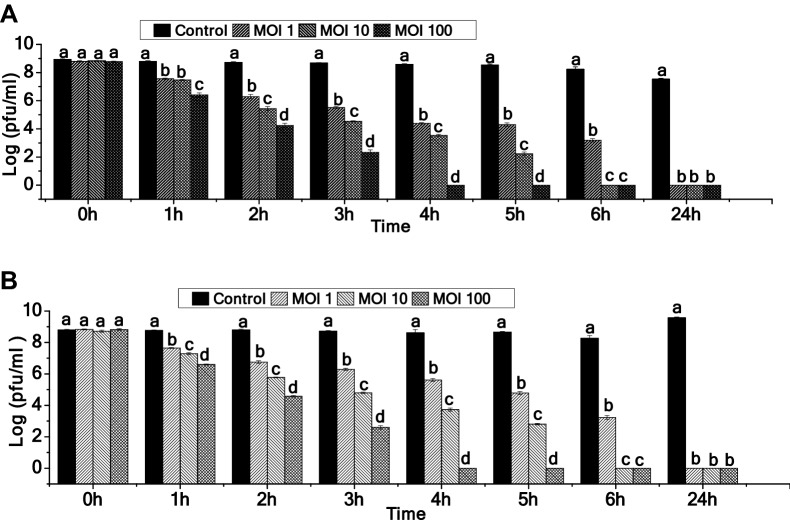
In-vitro assay of *Shigella* virus 2019SD1 against *S. dysenteriae* type 1 in (**A**) Saline and (**B**) Biodigester effluent. Error bar represents SEM (n = 3). Values of a given phage depicted as column without common letter differ significantly at *p *≤ 0.05 as per the Tukey’s HSD test.

The data on bactericidal effect of 2019SD1 on overnight-grown cultures of *S. dysenteriae* type 1 under saline conditions suggested that the growth of bacteria was significantly retarded even at MOI:1 within 1 h of incubation (p ≤ 0.05 as per Tukey’s HSD test). Furthermore, 5.61 log-reduction in bacterial growth compared to control was noticed at MOI:1 within 6 h of incubation and, at 24 h of incubation no bacterial growth was observed. In case of MOI:10 and MOI:100, bacterial growth was completely inhibited within 6 h and 4 h of incubation, respectively (Fig. 2A). When the host lysis test was performed using sterilized Biodigester effluent, similar results were obtained (Fig. 2B). Data on efficiency of plating (EOP) suggested that phage 2019SD1 exhibited lytic activity against multiple hosts including *S. dysenteriae*, *E. coli, V. cholerae, E. saccharolyticus* and *E. faecium* with a varying degree of EOP (Table 1)*.* Different host ranges have been reported for *Shigella* spp. infected with phages of *Drexlerviridae*. Wichels et al.^57^ stated that *Siphoviridae* (now known as *Drexlerviridae*) phages should be considered only as restricted host range phages. This was confirmed in another study which reported that pSf-2 phage produced clear plaques only in *S. flexneri* cultures (ATCC 12022, 11 836 and 29903)^56^. Similarly, a more recently described phage (HCF1)^58^ belonging to *Drexlerviridae* family displayed lytic activity against two *Citrobacter* species (*C. freundii* and *C. amalanoticus*) but not against *S. dysenteriae.* In contrast, Hamdi et al.^55^ reported that *S. flexneri* and *E. coli* can be lysed by *Drexlerviridae* phage SH6 and Sfin-1. Furthermore, Ahamed et al.^29^ reported that *S. flexneri,* *S. dysenteriae*, *S. sonnei* and *E. coli* can be lysed by *Drexlerviridae* (formerly *Siphoviridae)* phage Sfin-1. Our data also clearly demonstrated that some *Drexlerviridae* phages can exhibit polyvalent properties. Moreover, regarding the fact that host range is one of the most important criteria in phage application^56^, 2019SD1 seems to be a potential phage as it has the capability to infect different species of *Shigella* that contributes to shigellosis outbreaks in addition to killing other bacterial pathogens.

### Phage stability under abiotic stress conditions

**Figure 3:**
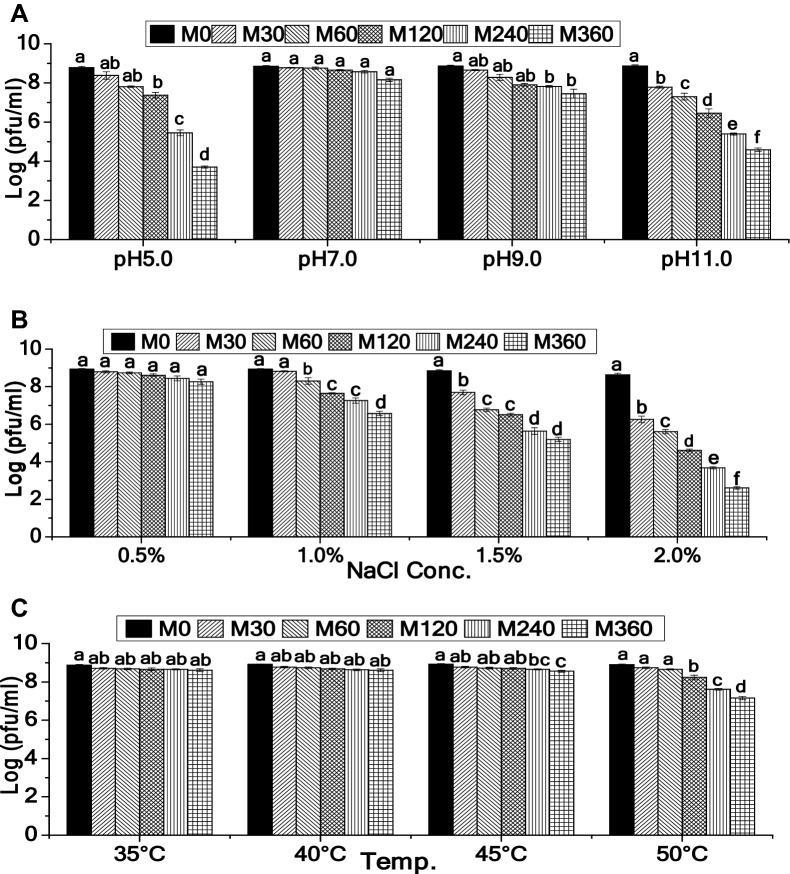
Stability of *Shigella* virus 2019SD1 under (**A**) Varying acidity and alkalinity levels, (**B**) Varying salinity (NaCl) concentrations and (**C**) Varying temperature regimes. Error bars represent SEM (n = 3). Values of a given phage depicted as column without common letter differ significantly at *p *≤ 0.05 as per the Tukey’s HSD test.

No significant reduction in the phage PFU was observed after 60 min of incubation at pH 5.0 (p > 0.05 as per Tukey’s HSD test). At pH 9.0, the phage was stable even upto 120 min of incubation. However, phage was highly unstable at pH 11.0, exhibiting significant reduction in PFU after 30 min of incubation (p ≤ 0.05 as per Tukey’s HSD test). Overall, after 360 min, 5.07, 1.41 and 4.29-log PFU reduction was noticed for the phage incubated at pH 5.0, 9.0 and 11.0 respectively (Fig. 3A). The phage showed high stability under pH values ranging from 7.0 to 9.0 while the titre of phage was slightly decreased with increasing acidity (pH 5.0) or alkalinity (pH 11.0). Compared to these findings, previously reported phages of *Shigella* such as pSf-1^52^, SH6^55^ and Sfin-1^29^ were infective under pH values ranging from 5 to 9, 5 to 11 and 5 to 12 respectively, but were less stable than 2019SD1. Contrastingly, compared to SfMu^25^ and SFPH2^59^, 2019SD1 was less stable.

Data on salinity tolerance suggested that there was no significant reduction in phage PFU even after 360 min at 0.5% NaCl treatment. At 1.0% NaCl treatment, phage was stable upto 30 min as evident from statistically insignificant reduction in PFU (p > 0.05 as per Tukey’s HSD test) and, only 2.36 log reduction in phage PFU was recorded after 360 min of incubation. Further enhancement in NaCl concentration had negative effect on phage PFU with 3.66 and 6.03 log reduction observed for 1.5% and 2.0% salt treatment, respectively (Fig. 3B). Data on thermal stability indicated that the phage was stable at 45 °C and 50 °C for 120 min and 60 min respectively as evident from insignificant change in PFU (p ≥ 0.05 as per Tukey’s HSD test). Overall, only 0.37 and 1.74 log reduction in phage PFU was recorded after 360 min of incubation at 45 °C and 50 °C (Fig. 3C). 2019SD1 was considerably stable under a wide range of temperature tested. On the contrary, *Shigella* phage psf-1 showed no loss of infectivity at temperature of 4 °C, 20 °C and 25 °C but when temperature started rising from 25 to 30 °C, 37 °C and 50 °C, phage infectivity showed a decreasing trend^53^.

### Preservation of phage

Data on viability of phages stored at varying temperature regimens suggested that there was a progressive and statistically significant reduction in PFU at − 20 °C. In case of storage at − 80 °C phage PFU was stable only upto 15 days. On the contrary, storage at 4 °C provided statistically stable phage count even after 75 days. Storage at 4 °C and 25 °C for 90 days resulted into loss of phage PFU upto 1.39 and 4.39 log respectively compared to initial titer. On the other hand, storage at − 20 °C and − 80 °C for 90 days resulted in considerable loss of phage PFU that was as high as 4.49 and 5.27log respectively when compared with initial titer values (Table 2).

**Table 2:** Viability of *Shigella* virus 2019SD1 stored at varying temperature regimens.

Days	− 80 °C	− 20 °C	4 °C	25 °C
0	8.82 ± 0.11^a^	8.82 ± 0.11^a^	8.82 ± 0.11^a^	8.82 ± 0.11^a^
15	8.74 ± 0.02^a^	7.56 ± 0.13^b^	8.53 ± 0.19^a^	8.54 ± 0.10^a^
30	7.31 ± 0.16^b^	7.48 ± 0.17^b,c^	8.52 ± 0.26^a^	7.06 ± 0.06^b^
45	6.87 ± 0.09^b^	7.23 ± 0.12^b,c^	8.36 ± 0.12^a^	6.92 ± 0.03^b^
60	5.30 ± 0.17^c^	6.58 ± 0.29^c^	8.16 ± 0.09^a,b^	6.71 ± 0.10^b^
75	4.68 ± 0.05^d^	5.32 ± 0.24^d^	8.11 ± 0.08^a,b^	5.47 ± 0.15^c^
90	3.55 ± 0.08^e^	4.33 ± 0.18^e^	7.43 ± 0.22^b^	4.43 ± 0.22^d^

Values (PFU mL^−1^) of a given phage without common letter differ significantly at *p* ≤ 0.05 as per the Tukey’s HSD test. ± SEM (n=3).

### Genome characteristics of phage

**Figure 4:**
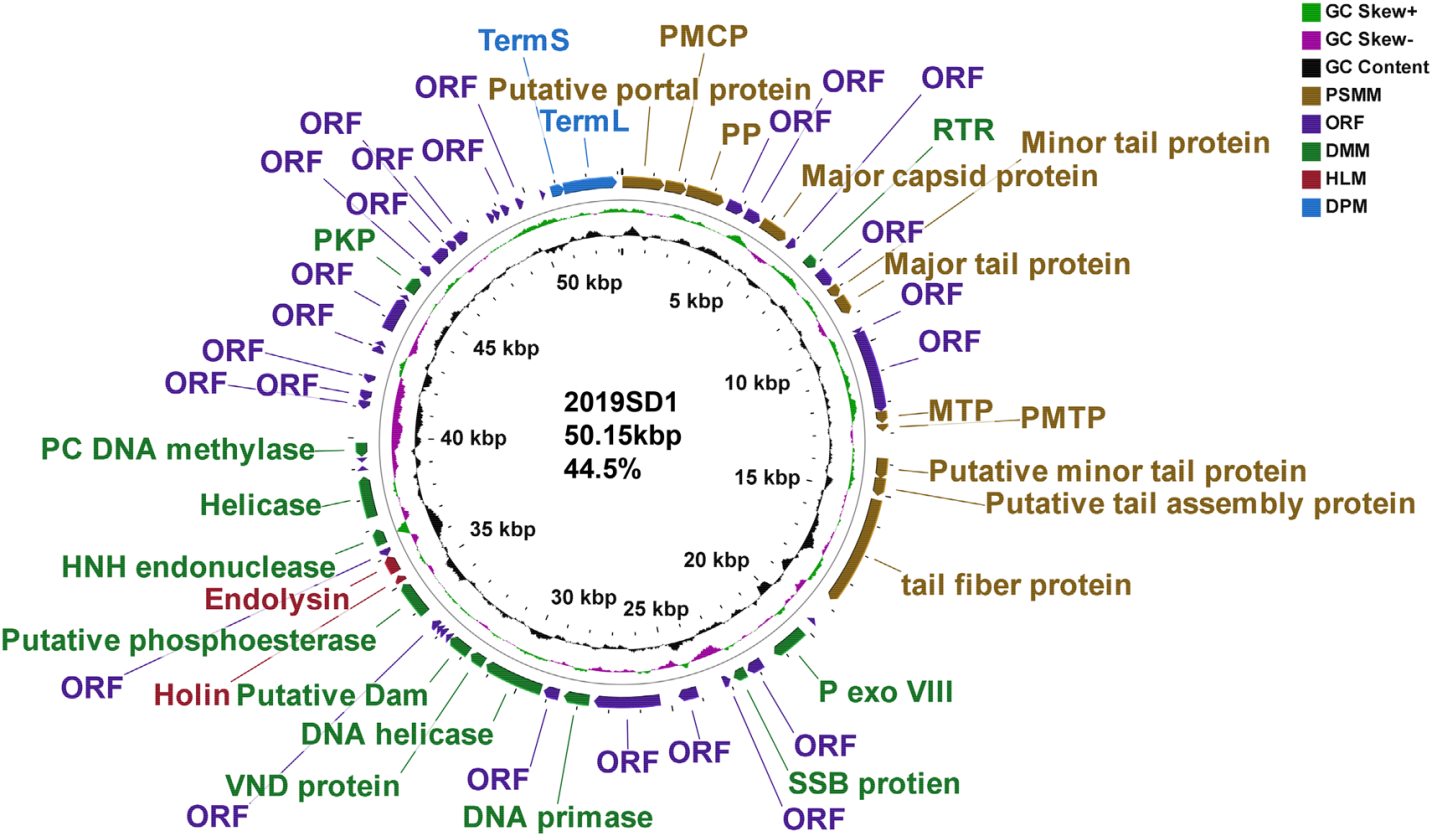
Genome organization of *Shigella* virus 2019SD1 as visualized using CGview software. ORFs with putative annotations are represented by specific colours as per their functional categories marked using arrows. *SSB protein* single strand DNA binding protein, *PC DM* Putative cytosine DNA methylase, *RTR* Ribonucleoside-triphosphate reductase, *P Exo VIII* Putative exodeoxyribonuclease VIII, *PKP* Putative polynucleotide 5′ kinase/3′ phosphatise, *VND protein* VRR-NUC domain-containing protein, *Putative Dam* Putative DNA *N*-6-adenine-methyltransferase, *MTP* Minor tail protein, *TermL* Large terminase subunit, *TermS* Small terminase subunit, *PMTP* Putative Minor tail protein.

DNAse I treatment of the nucleic acid of SD1 resulted into digestion of its genome whereas RNAse A treatment exhibited no sensitivity thereby revealing that 2019SD1 is a DNA virus. Nanopore sequencing of the phage DNA yielded a total of 47,654 raw reads with average and total read length of 1936 bp and 92,299,452 bp respectively. Nanopore assembly provided single scaffold of 73,606 bp (N50 value) which was used for subsequent analysis which revealed that 2019SD1 has a 53.15 Kb genome size with G + C content of 44.5%. The genome scanning for tRNA prediction suggested that the phage had no tRNA. The RAST analysis suggested a total of 77 ORFs in 2019SD1 genome (Fig. 4 and Supplementary Table S1). Further, a plausible Shine-Dalgarno sequence and start codons (56 with ATG, 12 with GTG and 9 with TTG) were identified. Sixty four putative 2019SD1 genes were found to be transcribed rightwards (on the genetic map), whereas thirteen genes transcribed leftwards (Fig. 4). The comparison of protein corresponding to each ORF with the non-redundant protein data bases using the NCBI BLASTp tool yielded matches with other proteins with identity values between 0 and 100% (Supplementary Table S1). The predicted ORFs were divided into four categories based on their functions. These categories included bacterial cell wall lysis proteins, structure and morphogenesis proteins, DNA metabolism proteins, and DNA packaging proteins. The detailed features of putative ORFs of phage 2019SD1, functional assignments, and homology to proteins in NCBI database are provided in supplementary data (Supplementary Tables S1 and S2).

### Biological properties of phage based on identified putative proteins

Results of functional annotation suggested the occurrence of putative proteins similar to holin (*ORF*41, 97.14% similarity with that of *Salmonella* phage slyngel), endolysin (*ORF*42, 68.04% similarity with that of *Escherichia* phage vojen), and Spanin (*ORF*43, 45.10% similarity with that of *Salmonella* virus STSR3) in 2019SD1 genome. Holin, unimolecular spanin and endolysin are critical for destruction of host cell during the burst step of phage life cycle. As soon as the new phage progeny is assembled, most of the phages lyse their host by recruiting a dual lysis system, which consists of a pore forming protein holin and a cell wall degrading enzyme endolysin or lysozyme. *ORF*41 and *ORF*42 were located contiguously in the middle part of the 2019SD1 genome that is involved in cell lysis similar to Sfin-1^29^.

RAST analysis followed by BLASTp confirmation and HHPred analysis indicated the occurrence of a protein similar to the tail fiber protein of *Escherichia* phage damhaus sharing 90.11% identity (accession number QHR69927.1). It is established that the key genes related to tail fiber protein (*ORF*21) could facilitate determination of threshold to get into the hosts through recognition of outer membrane receptors^56^. Results showed that the *ORF*21 of 2019SD1 shared considerable similarity (90.11%, 81% and 69%) with tail fiber protein of various phages like damhaus, Psf-1 and Sfin-1. This indicates the occurrence of diverse protein binding regions in 2019SD1 genome, a phenomenon which may contribute to determining the range of host species. Barbirz et al.^60^ also noticed that although the receptor-binding domains of three P22-like phages have no identifiable amino acid sequence similarity yet the host spectrum of the phage is different. Results of their study indicated that the interaction between the tail fiber protein and the host surface receptor happens through more specific binding. However, most of the studies have only investigated the conformation of tail fiber proteins and few studies have elaborated on the binding mechanism between the tail fiber proteins and the receptor on the outer membrane surface of host bacteria. Apparently, the mechanisms of host species determination and phage-receptor binding are far too complex and will attract attention of researchers for some time to come. Further research on these points may be useful for the development of artificial broad host range phages against multiple bacteria^59^. In the present study, the predicted protein of *ORF*20 was identified as the putative tail assembly protein owing to 99.49% identity with that of *Escherichia* phage egaa. Further, the major tail protein was found to be encoded by *ORF*12 with 96.08% identity to that of *Escherichia* phage haarsle. It is known that phages can alter their host spectrum by mutation of their tail fiber proteins^61^. Therefore, the sequence divergence in tail fiber proteins could yield different host specificities. The presence of multiple putative tail fiber proteins indicated the possibility of multivalent adsorption sites in 2019SD1. Interestingly, The product of *ORF*1 showed 69.12% similarity (E value: 0) with that of *Salmonella* phage slyngel (accession number QIN98153.1) that is considered to create the pore through which genome is packaged into the prohead. It is also a part of the packaging motor^62^.

The hypothetical proteins investigated through HHpred based analysis provided information about putative functions 11 ORFs which included various tail types of tail proteins and L-shaped tail fiber protein. It is notable that the L-shaped tail fibres in phage play important role not only in the initial recognition of certain *E. coli* host strains, binding to the O8- or O9-type O-antigen of the bacterial lipopolysaccharides but their presence increases the adsorption rate by a factor of 15^63^. In the present study, 2019SD1 successfully demonstrated the lytic activity against *E. coli* ATCC 25922 implying the presence of *E. coli* recognition mechanism and an effective adsorption through L-shaped tail fibres.

RAST analysis followed by BLASTp confirmation resulted in the identification of various putative proteins associated with DNA repair mechanism in the 2019SD1 genome. For instance, *ORF*33 (1965 bp, 654 aa) was found to encode for a protein similar to the putative exodeoxyribonuclease VIII of *Escherichia* phage egaa sharing 66.19% identity. It is reported that Exodeoxyribonuclease VIII (*ORF*23) facilitates in breaking double stranded DNA and degrading the genome at 5′ ends^64^. This results in straightening and repairing of the kinked and abnormal portions of a genome through homologous recombination. Notably, repairing of the genome mediated by exodeoxyribonuclease VIII works even in low-energy milieu as it does not need ATP to perform DNA repair^64^. It has been suggested that exodeoxyribonuclease VIII may enable the phages to remain stable in spite of UV-related mutations^64^. In the present study, the predicted proteins of *ORF*34 of 2019SD1 corresponding to VRR-NUC domain-containing protein shared 60.29% identity with that of *Escherichia* phage vojen. Notably, VRR-Nuc is a member of the primordial restriction endonuclease-like superfamily and is a part of FANCD2/FANCI-associated nuclease 1 (FAN1), a structure specific nuclease. FAN1 has been known to contribute in the repair of inter-strand DNA crosslinks like the ERCC1-XPF nuclease^65^. Predicted presence of putative Exodeoxyribonuclease VIII (*ORF*23) and VRR-NUC domain-containing protein (*ORF*34) in 2019SD1 genome indicates their utility in providing stability against probable DNA damage.

In the 2019SD1 genome, the putative DNA N-6-adenine-methyltransferase and cytosine DNA methylase were found to be encoded by *ORF*35 and *ORF*48 with 96.39% and 58.06% identity (E value: 8E−51) to that of *Escherichia* phage egaa *and Shigella* phage Sd1 respectively. Noticeably, the restriction endonucleases are part of restriction–modification (RM) system comprising of an endonuclease and a methyltransferase activity, with the main biological function to protect the host genome against foreign DNA, particularly the phage^66^. Features of *ORF*35 and *ORF*48 indicate the presence of DNA methylation mechanism to escape restriction modification system of host bacterium, a feature usually associated with polyvalent phages. Moreover, REBASE analysis suggested that 2019SD1 genomic DNA had no cleavage sites for type II restriction enzyme system *Eco*53K1 and *Eco*N1. It had only one site for *Eco*0109I and two sites each for *Eco*R1, *Eco*RV (Supplementary Fig. S1). The absence or limited number of cleavage sites in phage genome offers them advantage in terms of evading the restriction enzyme systems of host bacteria thereby potential increasing not only the survival ability and also the chances to infect other bacteria.

CRISPR (clustered regularly interspaced short palindromic repeat)-Cas adaptive immune systems are well-known defence mechanisms present in bacteria and archaea providing specific detection of neutralization of foreign nucleic acids such as phages^67^. In order to evade this, many phages develop anti-CRISPR systems through the expression of anti-CRISPR proteins^68,69^. In 2019SD1, *ORF*7 corresponding to NHis AcrE1 anti-CRISPR protein was found thereby indicating presence of an anti-CRISPR system. Further, the presence of a putative anti-repressor protein encoded by *ORF*32 in 2019SD1 suggests its lytic life cycle as this protein is required for the switch from the lysogenic to the lytic life cycle^70^. In addition, no ORFs were identified corresponding to excisionase, integrase, or repressor genes in the genome of 2019SD1. These observations unequivocally indicate that the phage 2019SD1 has a lytic lifecycle. Another noticeable feature of 2019SD1 genome was identification of putative Anti-restriction endonuclease (*ORF*71) which is known to participate in viral anti-host defense system through its DNA mimicking properties^71^. Overall, occurrence of a battery of various putative proteins associated with anti-host mechanisms as discussed above in conjunction with data on in vitro studies indicates the polyvalent nature of phage 2019SD1.

### Prediction of conserved domains

Based on data analysis using Pfam^43^ and NCBI Web CD-search tool^44^, at least three conserved domains were identified which were related to *ORF*15 (COG5281 superfamily), *ORF*31 (zf-CHC2 superfamily) and *ORF*39 (PLATZ superfamily). NCBI Web CD-search tool suggested that COG5281 domain is a Phage-related minor tail protein and the only member of the superfamily cl34971 whereas zf-CHC2 domain is mainly involved in DNA binding in DNA primases. Detailed features of the conserved domains are provided in Supplementary Table S6.

### Comparison of 2019SD1 with other phages of Drexlerviridae family

#### Comparative genomic analysis

**Figure 5:**
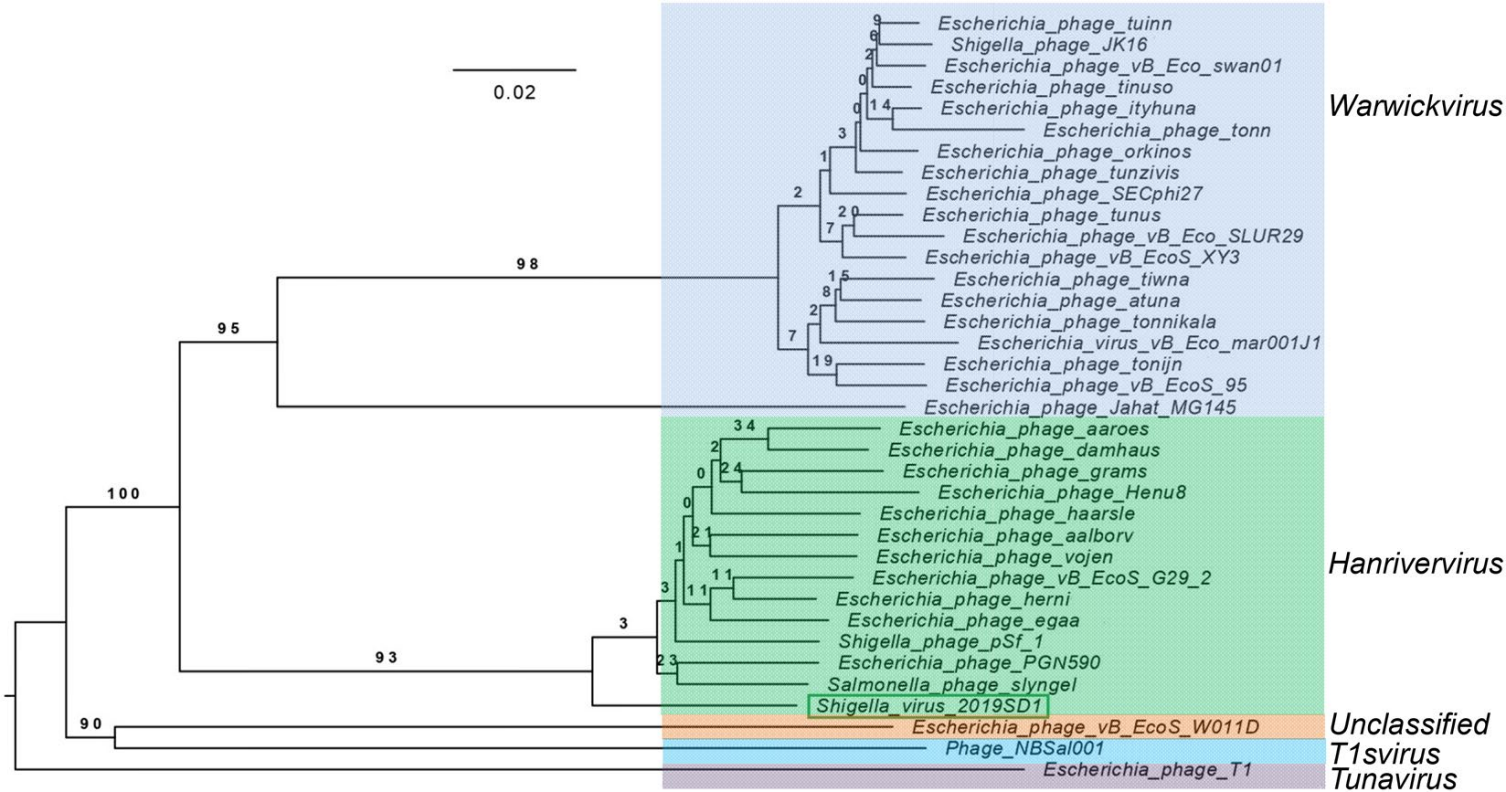
Comparison of the whole genome of *Shigella* virus 2019SD1 with 34 other members of *Drexlerviridae* using Phylogenomic Genome-BLAST Distance Phylogeny (GBDP) tree with prokaryotic viruses as default setting. Tree was generated using VICTOR.

**Figure 6:**
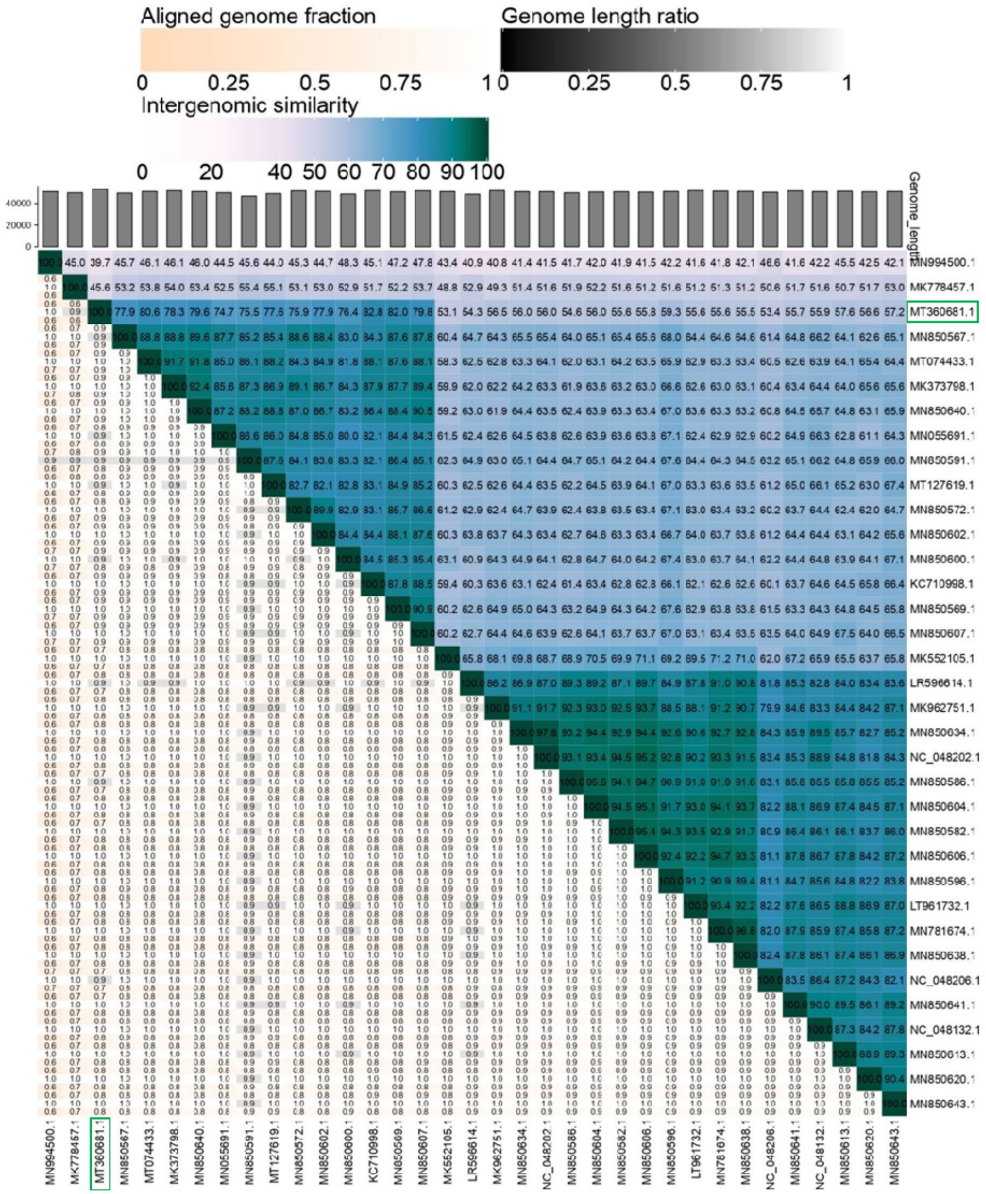
Heat map showing whole genome comparison of *Shigella* virus 2019SD1 with 34 other members of *Drexlerviridae*. Heat map was generated using VIRIDIC server with the threshold set to 95% for species differentiation.

The genome sizes, GC content and ORFs of all *Drexlerviridae* phages used for the comparative analysis ranged from ~ 44.6 Kb (phage orkinos) to 51.85 Kb (Phage JK16), 42.5% (Phage NBSal001) to 49.8% (phage orkinos), and 51 ORFs (phage PGN590) to 89 ORFs (virus 95), respectively (Supplementary Table S3). Genome-BLAST Distance Phylogeny (GBDP) tree generated using VICTOR clearly placed 2019SD1 in a separate clade of 13 phages which belonged to the genus *Hanrivervirus* of subfamily *Tempevirinae* (Fig. 5). Furthermore, the heat map generated using VIRIDIC server suggested that 2019SD1 showed maximum identity (83%) with the phage pSf-1 which is a member of *Tempevirinae* subfamily in *Drexlerviridae* family having similarity with the genus *Hanrivervirus* (Fig. 6)*.* Nevertheless, its functional modules differed considerably with five closely related phagse as suggested through linear comparison figures of multiple genomic loci drawn using EasyFig (Supplementary Fig. S2).The Bacterial and Archaeal Viruses Subcommittee (BAVS) describes a genus as a cohesive group of viruses sharing the nucleotide sequence similarity of > 70, with two viruses belonging to the same species differing by less than 5% from each other at the nucleotide level according to BLASTn data^51^. Accordingly, *Shigella* virus 2019SD1 exhibited similarity with the genus *Hanrivervirus* of *Tempevirinae* subfamily under *Drexlerviridae* family.

To date, a total of 76 complete genome sequences of *Shigella* phage are available in the NCBI database. The sizes of their genome range between 39.8 Kb and 170.7 Kb. According to the classification standards of the ICTV (International Committee on Taxonomy of Viruses)^72,73^, they all belong to the order Caudovirales. At the family level, there are 44, 14, 6, 4, 3, 2, 2 and 1 *Shigella* phages belonging to the families *Myoviridae, Drexlerviridae, Podoviridae, Autographiviridae, Siphoviridae, Demerecviridae, Ackermannviridae* and *Microviridae,* respectively.

### Comparative analysis of protein phylogeny

**Figure 7:**
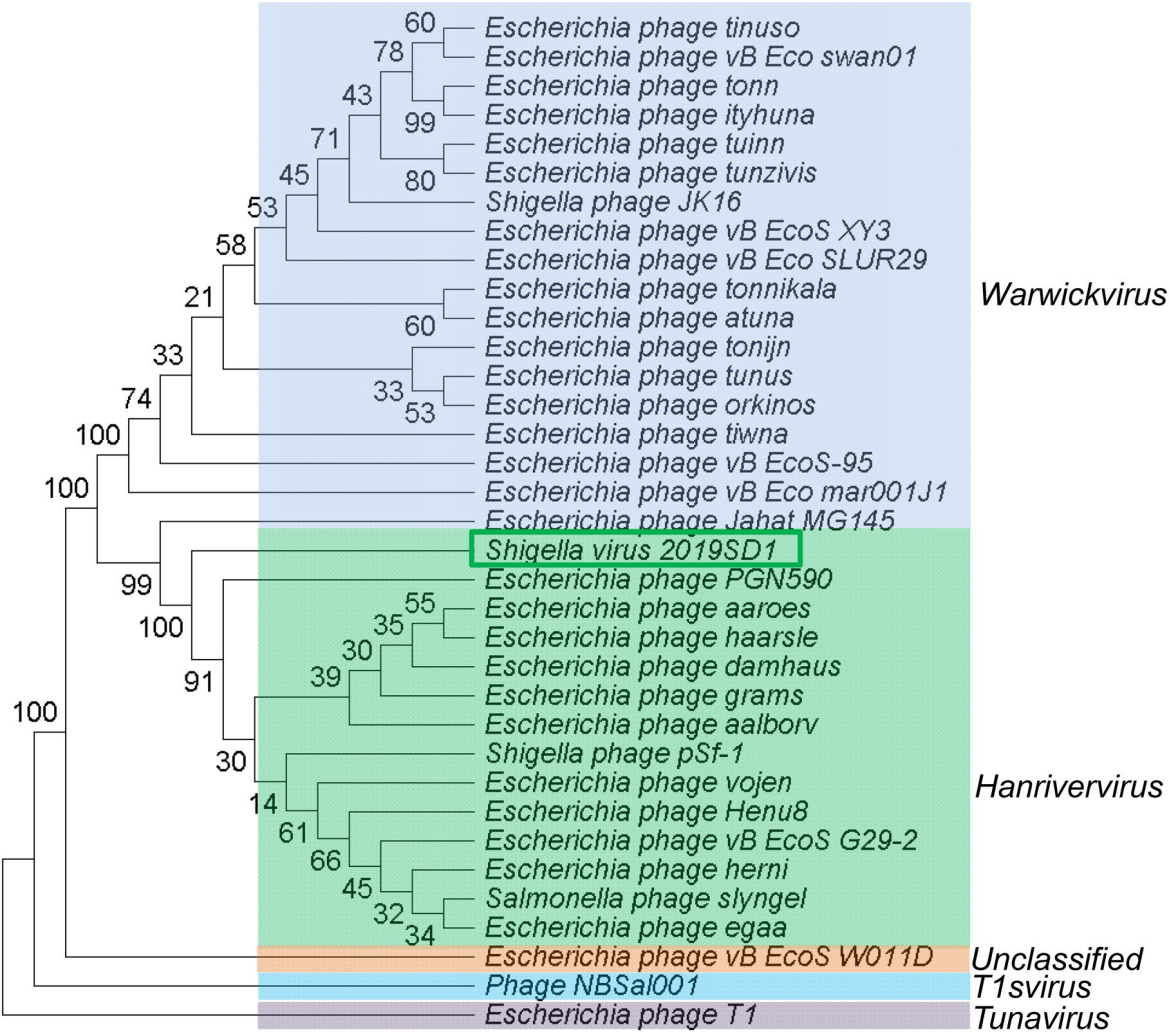
Comparison of the concatenated protein phylogeny of *Shigella* virus 2019SD1 with 34 other members of *Drexlerviridae* taking into account four individual marker genes (helicase, primase, portal protein and large terminase subunit). Protein data were concatenated using Geneious Prime 2020.2.2 (Build 2020-07-23 08:02 Java Version 11.0.4 + 11 64 bit) and evolutionary analysis was carried out using the maximum parsimony method in MEGA X^49^. The percentage of replicate trees in which the associated taxa clustered together in the bootstrap test (1000 replicates) is displayed next to the branches^76^.

The BLASTp search with predicted ORFs of 2019SD1 genome indicated that its structural genes and replication machinery were more similar to those of *Drexlerviridae* family compared to the phages of any other family (Supplementary Table S1). Furthermore, CoreGenes analysis suggested that the 59 putative proteins encoded were common among five phages, showing coverage upto 77.63%, 62.77%, 73.75%, 73.75% and 71.08% of protein profiles of 2019SD1, pSf-1, slyngel, vojen and herni phages, respectively (Supplementary Table S5). It has been suggested that phages can be grouped together when they share ≥ 40% of core proteins with each other^74,75^. According to this cut-off value, 2019SD1 may be grouped in the genus *Hanrivervirus* along with these five phages. The analysis also indicted that these six phages including 2019SD1 shared high homology with each other at protein level. Protein sequences of 34 phages, out of 35 chosen in detailed genomic analysis, were retrieved from NCBI database and were taken further for carrying out phylogenetic analysis using MEGA X software. Details of one phage namely *Escherichia* phage SECphi27 (NCBI accession number LT961732) were not available in the database, hence it could not be included in the phylogenetic analysis based on the protein sequences. Further, the comparison of the concatenated protein phylogeny of 2019SD1 with that of 34 members of *Drexlerviridae* taking into account four individual marker genes (helicase, primase, portal protein and large terminase subunit) was carried out which also suggested that 2019SD1 formed a distinct clade with the closest match of group of the taxa belonging to the genus *Hanrivervirus* (Fig. 7)*.*

## Conclusions

Shigellosis affects a large population worldwide as evident from the considerable numbers of episodes reported from various countries. In the current study, we have described the detailed biological properties and comparative whole genomic analysis of 2019SD1, a polyvalent *Shigella* phage exhibiting lytic activity against *S. dysenteriae*, *E. coli, V. cholerae, E. saccharolyticus* and *E. faecium.* Further, based on the comparative analysis of the detailed morphological features, whole genome sequences and predicted individual marker gene sequences (DNA Primase, ATP Dependent DNA Helicase, Large Terminase Protein and Portal Protein) with other known phage sequences, we establish its identity as 2019SD1 *Shigella* virus belonging to the genus *Hanrivervirus* of subfamily *Tempevirinae* under the family *Drexlerviridae*. The genome analysis data indicate the occurrence of putative tail fiber proteins suggesting the possibility of multivalent adsorption sites in 2019SD1. In addition, an ORF specific to DNA methylation mechanism was also reported that may have role in escaping restriction modification system of host bacterium. Moreover, occurrence of putative anti-CRISPR and anti-restriction endonulcease systems suggest the well-established anti-host defence system in 2019SD1 indicating its biocontrol potential. The present study is the first report of a polyvalent lytic phage which is active against five of the most prevalent bacterial pathogens of *Enterobacteriaceae* family and therefore might have a significant role not only in developing phage-based therapy against the Shigellosis, but also in unearthing the various evolutionary aspects and host lytic spectra of phages.

## Supplementary Information

Supplementary Information.
