# Supplementary material for: Isolation, phenotypic characterization and comparative genomic analysis of 2019SD1, a polyvalent enterobacteria phage
Source: Sci Rep. 2021 Nov 12;11:22197. doi: 10.1038/s41598-021-01419-8 (PMC8590004; doi:10.1038/s41598-021-01419-8)
Supplement: Supplementary file 1 — Supplementary Information. [file 41598_2021_1419_MOESM1_ESM.docx]

**Supplementary data (Table S1 to S6 and Figures S1 to S2)**

**MS Title: Isolation, phenotypic characterization and comparative genomic analysis of 2019SD1, a polyvalent enterobacteria phage**

Prince Kumar**^♣$^**, Mukesh K. Meghvansi**^♣#^**, D. V. Kamboj**^♣^***

*^♣^Biotechnology Division, Defence Research & Development Establishment, Gwalior 474002, Madhya Pradesh, India*

*^$^Current Address: Regional Ayurveda Research Institute, Gwalior 474009, Madhya Pradesh, India*

*^#^Current Address: Bioprocess Technology Division, Defence Research & Development Establishment, Gwalior 474002, Madhya Pradesh, India*

**Corresponding author (E-mail:* [*dvkamboj@gmail.com*](mailto:dvkamboj@gmail.com)*)*

**Supplementary Table** S**1.**  General features of putative ORFs of phage 2019SD1, functional assignments, and homology to proteins in NCBI database.

| **ORF** | **Locus Tag** | **Coordinate** | **Strand** | **aa*** | **Predicted function, phage name and Closest protein match with accession number** | **E value** | **% identity** |
| --- | --- | --- | --- | --- | --- | --- | --- |
| ORF1 | SD1_1 | 8…1375 | + | 455 | Putative portal protein (*Salmonella* phage slyngel),QIN98153.1 | 0 | 69.12 |
| ORF2 | SD1_2 | 1396…2103 | + | 235 | Putative minor capsid protein (*Escherichia* phage herni), QHR74790.1 | 3E-157 | 86.49 |
| ORF3 | SD1_3 | 2119…3384 | + | 421 | Prohead protease (*Escherichia* phage egaa), QHR70465.1 | 7E-178 | 78.76 |
| ORF4 | SD1_4 | 3518…4069 | + | 183 | Hypothetical protein pSf1_0066 (*Shigella* phage pSf-1), YP_008059788.1 | 4E-52 | 57.78 |
| ORF5 | SD1_5 | 4177…4707 | + | 176 | Hypothetical protein egaa_53 (*Escherichia* phage egaa), QHR70463.1 | 8E-67 | 89.57 |
| ORF6 | SD1_6 | 4777…5724 | + | 315 | Major capsid protein (*Escherichia* phage Henu8), QEA10079.1 | 0 | 86.58 |
| ORF7 | SD1_7 | 5861…6133 | + | 90 | Hypothetical protein pSf1_0069 (*Shigella* phage pSf-1), YP_008059791.1 | 8E-22 | 77.05 |
| ORF8 | SD1_8 | 6126…6569 | + | 147 | Hypothetical protein G292_00034 (*Escherichia* phage vB_EcoS_G29-2), QBQ81488.1 | 4E-45 | 92.31 |
| ORF9 | SD1_9 | 6634…7044 | + | 136 | Ribonucleoside-triphosphate reductase (*Escherichia* phage haarsle), QHR69622.1 | 8E-47 | 88.24 |
| ORF10 | SD1_10 | 7232…7816 | + | 194 | Hypothetical protein SP36_28 (*Salmonella* phage 36), YP_009223450.1 | 9E-68 | 61.18 |
| ORF11 | SD1_11 | 7866…8249 | + | 127 | Minor tail protein (*Escherichia* phage herni), QHR74799.1 | 1E-24 | 96.08 |
| ORF12 | SD1_12 | 8287…8910 | + | 207 | Major tail protein (*Escherichia* phage haarsle), QHR69625.1 | 1E-145 | 96.08 |
| ORF13 | SD1_13 | 9044…9412 | + | 122 | Hypothetical protein pSf1_0075 (*Shigella* phage pSf-1), YP_008059797.1 | 5E-72 | 100 |
| ORF14 | SD1_14 | 9480…9632 | + | 50 | Hypothetical protein G292_00040 (*Escherichia* phage vB_EcoS_G29-2), QBQ81494.1 | 6E-18 | 82.5 |
| ORF15 | SD1_15 | 9608…12286 | + | 892 | Hypothetical protein grams_44 (*Escherichia* phage grams), QHR65235.1 | 0 | 60.37 |
| ORF16 | SD1_16 | 12276…12659 | + | 127 | Minor tail protein (*Escherichia* phage Jahat_MG145), QBZ71363.1 | 1E-07 | 88.89 |
| ORF17 | SD1_17 | 12691…12930 | + | 79 | Putative minor tail protein (*Escherichia* phage grams), QHR65236.1 | 1E-18 | 100 |
| ORF18 | SD1_18 | 12991…13752 | + | 253 | Hypothetical protein pSf1_0079 (*Shigella* phage pSf-1), YP_008059801.1 | 0 | 100 |
| ORF19 | SD1_19 | 13796…14464 | + | 222 | Putative minor tail protein (*Escherichia* phage herni), QHR74805.1 | 1E-138 | 82.3 |
| ORF20 | SD1_20 | 14445…15041 | + | 198 | Putative tail assembly protein (*Escherichia* phage egaa), QHR70450.1 | 1E-140 | 99.49 |
| ORF21 | SD1_21 | 15163…18771 | + | 1202 | Tail fiber protein (*Escherichia* phage damhaus), QHR69927.1 | 0 | 90.11 |
| ORF22 | SD1_22 | 19728…19564 | - | 264 | Hypothetical protein pSf2_020 (*Shigella* phage pSf-2), YP_009112958.1 | 2E-10 | 70 |
| ORF23 | SD1_23 | 20142…21314 | + | 390 | Putative exodeoxyribonuclease VIII (*Escherichia* phage egaa), QHR70445.1 | 3E-151 | 66.19 |
| ORF24 | SD1_24 | 21396…21674 | + | 92 | Hypothetical protein G292_00051 (*Escherichia* phage vB_EcoS_G29-2), QBQ81505.1 | 3E-57 | 96.74 |
| ORF25 | SD1_25 | 21805…22338 | + | 177 | Hypothetical protein pSf1_0087 (*Shigella* phage pSf-1), YP_008059809.1 | 8E-100 | 99.35 |
| ORF26 | SD1_26 | 22410…22841 | + | 143 | Single-stranded DNA binding protein (*Escherichia* phage egaa), QHR70442.1 | 3E-26 | 87.72 |
| ORF27 | SD1_27 | 23206…22949 | - | 85 | Hypothetical protein pSf1_0089 (*Shigella* phage pSf-1), YP_008059811.1 | 1E-52 | 98.82 |
| ORF28 | SD1_28 | 23843…23193 | - | 216 | Hypothetical protein vojen_56 (*Escherichia* phage vojen), QHR65377.1 | 4E-117 | 98.77 |
| ORF29 | SD1_29 | 24102…24737 | + | 211 | Hypothetical protein herni_2 (*Escherichia* phage herni), QHR74735.1 | 1E-35 | 100 |
| ORF30 | SD1_30 | 25338…27494 | + | 718 | Hypothetical protein G292_00058 (*Escherichia* phage vB_EcoS_G29-2), QBQ81512.1 | 6E-101 | 78.67 |
| ORF31 | SD1_31 | 27643…28485 | + | 280 | DNA primase (*Escherichia* phage haarsle), QHR69645.1 | 5E-74 | 97.46 |
| ORF32 | SD1_32 | 28646…29158 | + | 170 | Hypothetical protein pSf1_002 (*Shigella* phage pSf-1), YP_008059724.1 | 1E-61 | 100 |
| ORF33 | SD1_33 | 29242…31206 | + | 654 | DNA helicase (*Escherichia* phage vojen), QHR65383.1 | 0 | 65.18 |
| ORF34 | SD1_34 | 31299…31808 | + | 169 | VRR-NUC domain-containing protein (*Escherichia* phage vojen), QHR65384.1 | 1E-42 | 60.29 |
| ORF35 | SD1_35 | 31901…32653 | + | 250 | Putative DNA N-6-adenine-methyltransferase (*Escherichia* phage egaa), QHR70432.1 | 1E-118 | 96.39 |
| ORF36 | SD1_36 | 32650…32832 | + | 60 | Hypothetical protein egaa_21 (*Escherichia* phage egaa), QHR70431.1 | 4E-11 | 96.55 |
| ORF37 | SD1_37 | 32880…33074 | + | 64 | No significant similarity found | - | - |
| ORF38 | SD1_38 | 33046…33225 | + | 59 | Hypothetical protein pSf1_008 (*Shigella* phage pSf-1), YP_008059730.1 | 2E-34 | 96.61 |
| ORF39 | SD1_39 | 33215…33457 | + | 80 | Hypothetical protein pSf1_009 (*Shigella* phage pSf-1), YP_008059731.1 | 1E-50 | 98.75 |
| ORF40 | SD1_40 | 33807…34997 | + | 396 | Putative phosphoesterase (*Escherichia* phage grams), QHR65261.1 | 3E-97 | 78.28 |
| ORF41 | SD1_41 | 35082…35327 | + | 81 | Holin (*Salmonella* phage slyngel), QIN98193.1 | 4E-40 | 97.14 |
| ORF42 | SD1_42 | 35445…36026 | + | 193 | Endolysin (*Escherichia* phage vojen), QHR65391.1 | 1E-55 | 68.06 |
| ORF43 | SD1_43 | 36277…36038 | - | 79 | Hypothetical protein tonnikala_12 (*Escherichia* phage tonnikala), QHR71283.1 | 8E-35 | 95.16 |
| ORF44 | SD1_44 | 36448…36978 | + | 176 | HNH endonuclease (*Escherichia* phage tonnikala), QHR71284.1 | 6E-55 | 91.00 |
| ORF45 | SD1_45 | 37392…38747 | + | 451 | Helicase (*Citrobacter* virus HCF1), QGF21262.1 | 2E-75 | 62.69 |
| ORF46 | SD1_46 | 38960…39076 | + | 38 | No significant similarity found | - | - |
| ORF47 | SD1_47 | 39342…39220 | - | 40 | No significant similarity found | - | - |
| ORF48 | SD1_48 | 39845…39387 | - | 152 | Putative cytosine DNA methylase (*Shigella* phage Sd1),EET0315114.1 | 8E-51 | 58.06 |
| ORF49 | SD1_49 | 40150…39842 | - | 102 | No significant similarity found | - | - |
| ORF50 | SD1_50 | 40490…40377 | - | 37 | Hypothetical protein grams_3 (*Escherichia* phage grams), QHR65194.1 | 3E-11 | 89.19 |
| ORF51 | SD1_51 | 40763…40590 | - | 57 | Hypothetical protein aaroes_14 (*Escherichia* phage aaroes), QHR65729.1 | 4E-25 | 100 |
| ORF52 | SD1_52 | 41219…40938 | - | 93 | Hypothetical protein aaroes_15 (*Escherichia* phage aaroes), QHR65730.1 | 1E-56 | 92.47 |
| ORF53 | SD1_53 | 41551…41219 | - | 110 | Hypothetical protein slyngel_62 (*Salmonella* phage slyngel), QIN98205.1 | 1E-36 | 95.31 |
| ORF54 | SD1_54 | 41756…41565 | - | 63 | Hypothetical protein G292_00084 (*Escherichia* phage vB_EcoS_G29-2), QBQ81538.1 | 1E-36 | 95.24 |
| ORF55 | SD1_55 | 42075…41803 | - | 90 | Hypothetical protein G292_00085 (*Escherichia* phage vB_EcoS_G29-2),QBQ81539.1 | 6E-58 | 96.67 |
| ORF56 | SD1_56 | 42834…43085 | + | 83 | Hypothetical protein damhaus_51 (*Escherichia* phage damhaus), QHR69966.1 | 1E-45 | 97.4 |
| ORF57 | SD1_57 | 43090…43257 | + | 55 | Hypothetical protein pSf1_0034 (*Shigella* phage pSf-1), YP_008059756.1 | 4E-18 | 95 |
| ORF58 | SD1_58 | 43647…44774 | + | 375 | Hypothetical protein tinuso_16 (*Escherichia* phage tinuso), QHR74093.1 | 4E-78 | 81.69 |
| ORF59 | SD1_59 | 44797…44949 | + | 50 | Hypothetical protein aalborv_48 (*Escherichia* phage aalborv), QHR68451.1 | 91.49 | 91.49 |
| ORF60 | SD1_60 | 45064…45609 | + | 181 | Putative polynucleotide 5' kinase/3' phosphatase (*Salmonella* phage slyngel), QIN98216.1 | 2E-123 | 96.05 |
| ORF61 | SD1_61 | 45606…45767 | + | 53 | Hypothetical protein egaa_73 (*Escherichia* phage egaa), QHR70483.1 | 5E-29 | 96.23 |
| ORF62 | SD1_62 | 45837…46133 | + | 98 | Hypothetical protein egaa_72 (*Escherichia* phage egaa), QHR70482.1 | 2E-46 | 81.63 |
| ORF63 | SD1_63 | 46130…46315 | + | 61 | Hypothetical protein slyngel_76 (*Salmonella* phage slyngel), QIN98219.1 | 6E-34 | 98.36 |
| ORF64 | SD1_64 | 46398…46946 | + | 182 | Hypothetical protein herni_42 (*Escherichia* phage herni), QHR74775.1 | 4E-99 | 93.88 |
| ORF65 | SD1_65 | 47006…47299 | + | 97 | No significant similarity found | - | - |
| ORF66 | SD1_66 | 47329…47778 | + | 149 | Hypothetical protein aaroes_35 (*Escherichia* phage aaroes), QHR65750.1 | 1E-77 | 88.06 |
| ORF67 | SD1_67 | 48008…48265 | + | 85 | Hypothetical protein pSf1_0051 (*Shigella* phage pSf-1), YP_008059773.1 | 9E-13 | 97.06 |
| ORF68 | SD1_68 | 48238…48525 | + | 95 | Hypothetical protein vojen_20 (*Escherichia* phage vojen), QHR65341.1 | 1E-64 | 100 |
| ORF69 | SD1_69 | 48600…48812 | + | 70 | Hypothetical protein slyngel_2 (*Salmonella* phage slyngel), QIN98145.1 | 8E-40 | 98.57 |
| ORF70 | SD1_70 | 48809…49018 | + | 69 | Hypothetical protein herni_48 (*Escherichia* phage herni), QHR74781.1 | 6E-20 | 93.18 |
| ORF71 | SD1_71 | 49112…49360 | + | 82 | Hypothetical protein SP126_00185 *(Salmonella* virus SP126), YP_009618013.1 | 5E-34 | 70.13 |
| ORF72 | SD1_72 | 49635…49868 | + | 77 | Hypothetical protein herni_50 *(Escherichia* phage herni), QHR74783.1 | 3E-45 | 97.18 |
| ORF73 | SD1_73 | 50009…50242 | + | 77 | Hypothetical protein vojen_25 (*Escherichia* phage vojen), QHR65346.1 | 1E-48 | 97.4 |
| ORF74 | SD1_74 | 50232…50384 | + | 50 | Hypothetical protein pSf1_0058 (*Shigella* phage pSf-1), YP_008059780.1 | 1E-27 | 100 |
| ORF75 | SD1_75 | 50463…50618 | + | 51 | Hypothetical protein slyngel_7 (*Salmonella* phage slyngel), QIN98150.1 | 8E-26 | 97.83 |
| ORF76 | SD1_76 | 50810…51250 | + | 146 | Terminase small subunit (*Escherichia* phage vojen), QHR65348.1 | 6E-100 | 97.95 |
| ORF77 | SD1_77 | 51228…52982 | + | 584 | Terminase large subunit (*Escherichia* phage Jahat_MG145), QBZ71379.1 | 3E-175 | 64.53 |

***Amino acid**

**Supplementary Table S2.** General features of hypothetical putative ORFs of phage 2019SD1, functional assignments, and distinct homologs proteins in HHpred analysis.

| **ORFs** | **PDB Entry**  **No.** | **Distinct homologs**  **Protein with phage** | **Probability**  **(%)** | **E-value** |
| --- | --- | --- | --- | --- |
| ORF4 | 6QYY_E | Capsid fiber protein; Decoration protein, *Bacillus* phage phi29 | 73.25 | 43 |
| ORF5 | 5WK1_S | Capsid Stabilizing Protein, *Pseudoalteromonas* phage TW1 | 98.01 | 0.00022 |
| ORF7 | 6AS3_D | NHis AcrE1 anti-CRISPR protein, *Pseudomonas* phage JBD5 | 30.68 | 42 |
| ORF8 | 3JVO_D | 13-membered ring,  Viral Protein, Enterobacteria phage HK97 | 91.99 | 0.9 |
| ORF13 | 2OB9_A | Tail assembly chaperone, Enterobacteria phage HK97 | 94.16 | 0.81 |
| ORF14 | 5LXL_A | Decoration protein, *Escherichia* phage T5 | 51.89 | 14 |
| ORF29 | 6F45_A | long tail fiber protein, *Salmonella* phage vB_SenM-S16, | 95.76 | 0. 057 |
| ORF30 | 4UW8_C | L-Shaped Tail Fiber Protein, Enterobacteria Phage T5 | 96.5 | 0.00069 |
| ORF32 | 5D50_K | Anti-repressor protein, *Salmonella* phage SPC32H | 98.04 | 0.000032 |
| ORF53 | 2KX4_A | Tail attachment protein, *Escherichia* virus Lambda | 24.78 | 210 |
| ORF 55 | 2C5R_C | Dna-Binding Protein/DNA, *Bacillus* virus phi29 | 70.37 | 26.78 |
| ORF 64 | 1DEK_A | Deoxynucleoside monophosphate kinase,  Enterobacteria phage T4 | 99.6 | 4.9e-14 |
| ORF69 | [6MNT_A](http://www.rcsb.org/pdb/explore/explore.do?structureId=6MNT) | Putative coat protein, Enterobacteria phage CUS-3 | 75.64 | 0.98 |
| ORF71 | [3WX4_A](http://www.rcsb.org/pdb/explore/explore.do?structureId=3WX4) | Anti-restriction endonuclease, Enterobacteria phage T4 | 24.43 | 28 |
| ORF 72 | 3PQI_A | Phage Baseplate, Iron-Binding protein, Bacteriophage phi92 | 31.59 | 54 |
| ORF73 | 5NGJ_B | Tail tube protein, *Escherichia* phage T5 | 43.97 | 35 |
| ORF74 | [2LMC_A](http://www.rcsb.org/pdb/explore/explore.do?structureId=2LMC) | Bacterial RNA polymerase inhibitor, Enterobacteria phage T7 | 72.45 | 7.3 |

**Supplementary Table** S**3.** Taxonomic and genomic features of phages investigated in this study.

| **S. No.** | **Accession number** | **Species** | **Genus** | **Subfamily*** | **Country** | **ORF** | **%GC** | **Size (kbp)** |
| --- | --- | --- | --- | --- | --- | --- | --- | --- |
| 1 | MT360681 | *Shigella* virus 2019SD1 | *Hanrivervirus* | *Tempevirinae* | India | 77 | 44.5 | 53.15 |
| 2 | MN850600 | *Escherichia* phage haarsle | *Hanrivervirus* | *Tempevirinae* | Denmark | 74 | 44.0 | 48.61 |
| 3 | MN055691 | *Escherichia* phage Henu8, | *Hanrivervirus* | *Tempevirinae* | China | 65 | 44.2 | 49.89 |
| 4 | MN850591 | *Escherichia* phage aalborv | *Hanrivervirus* | *Tempevirinae* | Denmark | 71 | 43.9 | 46.66 |
| 5 | MT127619 | *Escherichia* phage PGN590 | *Hanrivervirus* | *Tempevirinae* | India | 51 | 43.8 | 49.04 |
| 6 | MN850567 | *Escherichia* phage grams | *Hanrivervirus* | *Tempevirinae* | Denmark | 76 | 44.1 | 49.53 |
| 7 | MN850572 | *Escherichia* phage aaroes | *Hanrivervirus* | *Tempevirinae* | Denmark | 83 | 44.1 | 51.66 |
| 8 | MN850602 | *Escherichia* phage damhaus | *Hanrivervirus* | *Tempevirinae* | Denmark | 80 | 44.1 | 51.15 |
| 9 | MT074433 | *Salmonella* phage slyngel | *Hanrivervirus* | *Tempevirinae* | Denmark | 80 | 44.0 | 51.05 |
| 10 | MK373798 | *Escherichia* phage vB_EcoS_G29-2 | *Hanrivervirus* | *Tempevirinae* | Germany | 85 | 44.1 | 51.74 |
| 11 | MN850640 | *Escherichia* phage herni | *Hanrivervirus* | *Tempevirinae* | Denmark | 83 | 44.1 | 50.97 |
| 12 | KC710998 | *Shigella* phage pSf-1 | *Hanrivervirus* | *Tempevirinae* | Republic of Korea | 94 | 44.0 | 51.82 |
| 13 | MN850569 | *Escherichia* phage vojen | *Hanrivervirus* | *Tempevirinae* | Denmark | 80 | 44.1 | 50.71 |
| 14 | MN850607 | *Escherichia* phage egaa | *Hanrivervirus* | *Tempevirinae* | Denmark | 80 | 44.1 | 51.64 |
| 15 | MK552105 | *Escherichia* phage Jahat_MG145 | *Hanrivervirus* | *Tempevirinae* | Denmark | 85 | 45.7 | 50.98 |
| 16 | MN994500 | Phage NBSal001 | *Tlsvirus* | *Tempevirinae* | Peru | 78 | 42.5 | 50.92 |
| 17 | MN850596 | *Escherichia* phage tonn | *Warwickvirus* | *Tempevirinae* | Denmark | 86 | 44.5 | 51.01 |
| 18 | LT961732 | ***Escherichia* phage SECphi27 | *Warwickvirus* | *Tempevirinae* | USA | NA* | 44.7 | 51.81 |
| 19 | NC_048206 | *Escherichia* virus vB_Eco_mar001J1 | *Warwickvirus* | *Tempevirinae* | USA | 78 | 44.40 | 50.34 |
| 20 | NC_048132 | *Escherichia* virus 95 | *Warwickvirus* | *Tempevirinae* | Poland | [89](https://www.ncbi.nlm.nih.gov/genome/browse/#!/proteins/74989/434075\|Escherichia phage vB_EcoS-95/viral segment/) | 44.8 | 50.91 |
| 21 | MN781674 | *Escherichia* phage vB_EcoS_XY3 | *Warwickvirus* | *Tempevirinae* | China | 85 | 44.8 | 51.35 |
| 22 | MN850638 | *Escherichia* phage tunus | *Warwickvirus* | *Tempevirinae* | Denmark | 84 | 44.8 | 51.11 |
| 23 | MK962751 | *Shigella* phage JK16 | *Warwickvirus* | *Tempevirinae* | Netherlands | 84 | 44.5 | 51.85 |
| 24 | MN850634 | *Escherichia* phage tinuso | *Warwickvirus* | *Tempevirinae* | Denmark | 83 | 44.8 | 50.86 |
| 25 | NC_048202 | *Escherichia* phage vB_Eco_swan01 | *Warwickvirus* | *Tempevirinae* | UK | 83 | 44.7 | 50.87 |
| 26 | MN850586 | *Escherichia* phage orkinos | *Warwickvirus* | *Tempevirinae* | Denmark | 81 | 49.8 | 44.6 |
| 27 | MN850582 | *Escherichia* phage ityhuna | *Warwickvirus* | *Tempevirinae* | Denmark | [87](https://www.ncbi.nlm.nih.gov/genome/browse/#!/proteins/87168/769887\|Escherichia phage ityhuna/viral segment/) | 44.7 | 50.77 |
| 28 | MN850604 | *Escherichia* phage tunzivis | *Warwickvirus* | *Tempevirinae* | Denmark | [84](https://www.ncbi.nlm.nih.gov/genome/browse/#!/proteins/87213/769932\|Escherichia phage tunzivis/viral segment/) | 44.6 | 50.6 |
| 29 | MN850606 | *Escherichia* phage tuinn | *Warwickvirus* | *Tempevirinae* | Denmark | 82 | 44.7 | 50.51 |
| 30 | LR596614 | *Escherichia* phage vB_Eco_SLUR29 | *Warwickvirus* | *Tempevirinae* | UK | 78 | 44.47 | 48.47 |
| 31 | MN850641 | *Escherichia* phage tonijn | *Warwickvirus* | *Tempevirinae* | Denmark | [84](https://www.ncbi.nlm.nih.gov/genome/browse/#!/proteins/87206/769925\|Escherichia phage tonijn/viral segment/) | 44.6 | 51.63 |
| 32 | MN850620 | *Escherichia* phage atuna | *Warwickvirus* | *Tempevirinae* | Denmark | [84](https://www.ncbi.nlm.nih.gov/genome/browse/#!/proteins/87144/769863\|Escherichia phage atuna/viral segment/) | 44.6 | 50.73 |
| 33 | MN850613 | *Escherichia* phage tonnikala | *Warwickvirus* | *Tempevirinae* | Denmark | 83 | 44.8 | 51.28 |
| 34 | MN850643 | *Escherichia* phage tiwna | *Warwickvirus* | *Tempevirinae* | Denmark | 82 | 44.6 | 51.01 |
| 35 | MK778457 | *Escherichia* phage vB_EcoS_W011D | Unclassified | Unclassified | China | 85 | 46.2 | 49.85 |

******Tempevirinae* and unclassified phage belong to *Drexlerviridae* family as per the latest classification of ICTV updated in August 2020 (Last visited 02 October, 2021), **NA- information not available in NCBI database

**Supplementary Table S4.** Core Gene analysis of 2019SD1 phage with other similar Phage of genus *Hanrivervirus*.

|  | ***Shigella* virus 2019SD1 (**[**MT360681**](https://www.ncbi.nlm.nih.gov/nuccore/MT360681&doptcmdl=GenBank)**)** | ***Shigella* phage pSf-1 (**[**KC710998**](https://www.ncbi.nlm.nih.gov/nuccore/KC710998&doptcmdl=GenBank)**)** | ***Salmonella* phage slyngel (**[**MT074433**](https://www.ncbi.nlm.nih.gov/nuccore/MT074433&doptcmdl=GenBank)**)** | ***Escherichia* phage vojen (**[**MN850569**](https://www.ncbi.nlm.nih.gov/nuccore/MN850569&doptcmdl=GenBank)**)** | ***Escherichia* phage herni (**[**MN850640**](https://www.ncbi.nlm.nih.gov/nuccore/MN850640&doptcmdl=GenBank)**)** | ***Escherichia* phage egaa (**[**MN850607**](https://www.ncbi.nlm.nih.gov/nuccore/MN850607&doptcmdl=GenBank)**)** |
| --- | --- | --- | --- | --- | --- | --- |
| **S. No.** | **ORF name, Accession number** | **ORF name, Accession number** | **ORF name, Accession number** | **ORF name, Accession number** | **ORF name, Accession number** | **ORF name, Accession number** |
| 1 | Putative portal protein, QJT70530.1 | Hypothetical protein,AGI61445.1 | Putative portal protein,QIN98153.1 | Putative portal protein,QHR65350.1 | Putative portal protein,QHR74789.1 | Putative portal protein,QHR70467.1 |
| 2 | Putative minor capsid protein,  QJT70531.1 | Hypothetical protein,  AGI61447.1 | Head morphogenesis protein,  QIN98154.1 | Putative minor capsid protein,  QHR65351.1 | Putative minor capsid protein,  QHR74790.1 | Head morphogenesis protein,QHR70466.1 |
| 3 | prohead protease, QJT70532.1 | Hypothetical protein, AGI61448.1 | Major capsid protein,QIN98155.1 | Capsid proteinQHR65352.1 | Major capsid proteinQHR74791.1 | PRODUCT:prohead proteaseQHR70465.1 |
| 4 | Hypothetical protein, QJT70533.1 | Hypothetical protein,  AGI61449.1 | Putative zonula occludens toxin,  QIN98156.1 | Putative zonula occludens toxin,  QHR65353.1, | Putative zonula occludens toxin,QHR74792.1 | Putative zonula occludens toxin,QHR70464.1 |
| 5 | Hypothetical protein, QJT70534.1 | Hypothetical protein,  AGI61450.1 | Hypothetical protein,  QIN98157.1 | Hypothetical protein, QHR65354.1 | Hypothetical protein, QHR74793.1 | Hypothetical protein,QHR70463.1 |
| 6 | Major capsid protein, QJT70535.1 | Hypothetical protein,  AGI61451.1 | MCP protein, QIN98158.1 | MCP protein, QHR65355.1 | MCP protein, QHR74794.1 | MCP protein,QHR70462.1 |
| 7 | Hypothetical protein, QJT70536.1 | Hypothetical protein, AGI61452.1 | Hypothetical protein, QIN98159.1 | Hypothetical protein, QHR65356.1 | Hypothetical protein, QHR74795.1 | Hypothetical protein,QHR70461.1 |
| 8 | Hypothetical protein, QJT70537.1 | Hypothetical protein, AGI61453.1 | Hypothetical protein,QIN98160.1 | Hypothetical protein,QHR65357.1 | Hypothetical protein,QHR74796.1 | Hypothetical protein,QHR70460.1 |
| 9 | PRODUCT:ribonucleoside-triphosphate reductase, QJT70538.1 | Hypothetical protein, AGI61454.1 | Ribonucleoside-triphosphate reductase, QIN98161.1 | Ribonucleoside-triphosphate reductase, QHR65358.1 | Ribonucleoside-triphosphate reductase, QHR74797.1 | Ribonucleoside-triphosphate reductase,QHR70459.1 |
| 10 | Hypothetical protein, QJT70539.1 | Hypothetical protein,AGI61455.1 | Hypothetical protein, QIN98162.1 | Hypothetical protein, QHR65359.1 | Hypothetical protein, QHR74798.1 | Hypothetical protein,QHR70458.1 |
| 11 | Minor tail protein, QJT70540.1 | Hypothetical protein, AGI61456.1 | Minor tail protein, QIN98163.1 | Minor tail protein, QHR65360.1 | Minor tail protein, QHR74799.1 | Minor tail proteinQHR70457.1 |
| 12 | Hypothetical protein, QJT70541.1 | Hypothetical protein, AGI61457.1 | Putative major tail protein, QIN98164.1 | Putative major tail protein, QHR65361.1 | Major tail protein, QHR74800.1 | Major tail protein,QHR70456.1 |
| 13 | Major tail protein, QJT70542.1 | Hypothetical protein,AGI61458.1 | TfmS, QIN98165.1 | Tape measure chaperone, QHR65362.1 | TfmS,QHR74801.1 | fmS,QHR70455.1 |
| 14 | Hypothetical protein, QJT70544.1 | Hypothetical protein, AGI61460.1 | Hypothetical protein, QIN98166.1 | Hypothetical protein, QHR65363.1 | Hypothetical protein, QHR74802.1 | Hypothetical protein, QHR70454.1 |
| 15 | Putative minor tail protein, QJT70546.1 | Hypothetical protein, AGI61461.1 | Minor tail protein, QIN98167.1 | Putative minor tail protein, QHR65364.1 | Minor tail protein, QHR74803.1 | Putative minor tail protein,QHR70453.1 |
| 16 | Hypothetical protein, QJT70547.1 | Hypothetical protein, AGI61462.1 | Putative minor tail protein, QIN98168.1 | Putative minor tail protein, QHR65365.1 | Putative minor tail protein, QHR74804.1 | Putative minor tail protein,QHR70452.1 |
| 17 | Putative minor tail protein, QJT70548.1 | Hypothetical protein, AGI61463.1 | Putativ,e minor tail protein, QIN98169.1 | Putative minor tail protein, QHR65366.1 | Putative minor tail protein, QHR74805.1 | Putative minor tail protein, QHR70451.1 |
| 18 | Putative tail assembly protein, QJT70549.1 | Hypothetical protein, AGI61464.1 | Putative tail assembly protein, QIN98170.1 | Putative tail assembly protein, QHR65367.1 | Putative tail assembly protein, QHR74806.1 | Putative tail assembly protein, QHR70450.1 |
| 19 | Tail fiber protein, QJT70550.1 | Hypothetical protein, AGI61465.1 | Tail fiber protein, QIN98171.1 | Tail fiber protein, QHR65368.1 | Putative tail fiber protein, QHR74807.1 | Putative tail fiber protein, QHR70449.1 |
| 20 | Putative exodeoxyribonuclease VIII, QJT70552.1 | Hypothetical protein, AGI61468.1 | Putative exodeoxyribonuclease, VIIIQIN98175.1 | Putative exodeoxyribonuclease, VIIIQHR65372.1 | Putative exodeoxyribonuclease, VIIIQHR74811.1 | Putative exodeoxyribonuclease, VIIIQHR70445.1 |
| 21 | Hypothetical protein, QJT70553.1 | Hypothetical protein, AGI61469.1 | Hypothetical protein, QIN98176.1 | Hypothetical protein, QHR65373.1 | Hypothetical protein, QHR74812.1 | Hypothetical protein,QHR70444.1 |
| 22 | Hypothetical protein, QJT70554.1 | Hypothetical protein,AGI61470.1 | Putative recombination protein,QIN98177.1 | Putative recombination protein,QHR65374.1 | Putative recombination protein,QHR74813.1 | Putative recombination protein,QHR70443.1 |
| 23 | Single-stranded DNA binding protein, QJT70555.1 | Hypothetical protein, AGI61471.1 | Putative ssDNA-binding protein, QIN98178.1 | Putative ssDNA-binding protein, QHR65375.1 | Single-stranded DNA binding protein, QHR74814.1 | Single-stranded DNA binding protein, QHR70442.1 |
| 24 | Hypothetical protein, QJT70556.1 | Hypothetical protein, AGI61472.1 | Hypothetical protein, QIN98179.1 | Hypothetical protein, QHR65376.1 | Hypothetical protein, QHR74815.1 | Hypothetical protein, QHR70441.1 |
| 25 | Hypothetical protein, QJT70557.1 | Hypothetical protein, AGI61473.1 | Hypothetical protein, QIN98180.1 | Hypothetical protein, QHR65377.1 | TerL, QHR74816.1 | Hypothetical protein,QHR70440.1 |
| 26 | Hypothetical protein, QJT70558.1 | Hypothetical protein,AGI61475.1 | Hypothetical protein,QIN98182.1 | Hypothetical protein,QHR65379.1 | Hypothetical protein, QHR74735.1 | Hypothetical protein,QHR70438.1 |
| 27 | PRODUCT:DNA primase, QJT70560.1 | Hypothetical protein, AGI61384.1 | DNA primase, QIN98184.1 | DNA primase, QHR65381.1 | DNA primase, QHR74737.1 | PRODUCT:DNA primase ,QHR70436.1 |
| 28 | Hypothetical protein, QJT70561.1 | Hypothetical protein, AGI61385.1 | Putative transcriptional regulator, QIN98185.1 | Putative transcriptional regulator, QHR65382.1 | Putative transcriptional regulator, QHR74738.1 | Putative transcriptional regulator,QHR70435.1 |
| 29 | DNA helicase, QJT70562.1 | Hypothetical protein, AGI61386.1 | DNA helicase, QIN98186.1 | DNA helicase, QHR65383.1 | DNA helicase, QHR74739.1 | DNA helicase, QHR70434.1 |
| 30 | VRR-NUC domain-containing protein, QJT70563.1 | Hypothetical protein, AGI61388.1 | VRR-NUC domain-containing protein,QIN98187.1 | VRR-NUC domain-containing protein,QHR65384.1 | VRR-NUC domain-containing protein, QHR74740.1 | VRR-NUC domain-containing protein, QHR70433.1 |
| 31 | Putative DNA N-6-adenine-methyltransferase, QJT70564.1 | Hypothetical protein, AGI61389.1, | Putative DNA N-6-adenine-methyltransferase, QIN98188.1 | Putative DNA N-6-adenine-methyltransferase, QHR65385.1 | Putative DNA N-6-adenine-methyltransferase,QHR74741.1 | Putative DNA N-6-adenine-methyltransferase,QHR70432.1 |
| 32 | Hypothetical protein, QJT70565.1 | Hypothetical protein,AGI61390.1 | Hypothetical protein,QIN98189.1 | Hypothetical protein, QHR65386.1 | Hypothetical protein, QHR74742.1 | Hypothetical protein,QHR70431.1 |
| 33 | Hypothetical protein, QJT70567.1 | Hypothetical protein, AGI61391.1 | Hypothetical protein, QIN98190.1 | Hypothetical protein, QHR65387.1 | Hypothetical protein, QHR74743.1 | Hypothetical protein,QHR70430.1 |
| 34 | Hypothetical protein, QJT70568.1 | Hypothetical protein,AGI61392.1 | Hypothetical protein,QIN98191.1 | Hypothetical protein,QHR65388.1 | Hypothetical protein,QHR74744.1 | Hypothetical protein,QHR70429.1 |
| 35 | Putative phosphoesterase, QJT70569.1 | Hypothetical protein,AGI61393.1 | Putative phosphoesterase,QIN98192.1 | Putative phosphoesterase, QHR65389.1 | Putative phosphoesterase,QHR74745.1 | Putative phosphoesterase,QHR70428.1 |
| 36 | Holin, QJT70570.1 | Hypothetical protein, AGI61394.1 | Holin, QIN98193.1 | Holin, QHR65390.1 | Holin, QHR74746.1 | Holin, QHR70427.1 |
| 37 | Endolysin, QJT70571.1 | Hypothetical protein,AGI61395.1 | Endolysin,QIN98194.1 | Endolysin,QHR65391.1 | Putative lysozyme, QHR74747.1 | Putative lysozyme,QHR70426.1 |
| 38 | PRODUCT: helicase, QJT70574.1 | Hypothetical protein, AGI61401.1 | Putative helicase, QIN98198.1 | Putative helicase, QHR65394.1 | Putative helicase, QHR74751.1 | Putative helicase, QHR70422.1 |
| 39 | Putative cytosine DNA methylase, QJT70576.1 | Hypothetical protein, AGI61404.1 | Putative DNA methylase,QIN98201.1 | Putative site specific DNA methylase , QHR65397.1 | Putative site specific DNA methylase,QHR74754.1 | Putative site specific DNA methylase,QHR70419.1 |
| 40 | Hypothetical protein, QJT70579.1 | Hypothetical protein, AGI61407.1 | Hypothetical protein,QIN98203.1 | Hypothetical protein,QHR65399.1 | Hypothetical protein,QHR74756.1 | Hypothetical protein, QHR70437.1 |
| 41 | Hypothetical protein, QJT70581.1 | Hypothetical protein, AGI61409.1 | Hypothetical protein, QIN98205.1 | Hypothetical protein, QHR65322.1 | Hypothetical protein, QHR74758.1 | Hypothetical protein,QHR70417.1 |
| 42 | Hypothetical protein, QJT70582.1, | Hypothetical protein, AGI61413.1 | Hypothetical protein, QIN98207.1 | Hypothetical protein, QHR65324.1 | Hypothetical protein, QHR74762.1 | Hypothetical protein,QHR70415.1 |
| 43 | Hypothetical protein, QJT70583.1 | Hypothetical protein, AGI61414.1 | Hypothetical protein, QIN98208.1 | Hypothetical protein, QHR65325.1 | Hypothetical protein, QHR74763.1 | Hypothetical protein, QHR70411.1 |
| 44 | Hypothetical protein, QJT70584.1 | Hypothetical protein, AGI61416.1 | Hypothetical protein, QIN98210.1 | Hypothetical protein, QHR65326.1 | Hypothetical protein, QHR74765.1 | Hypothetical protein,QHR70487.1 |
| 45 | Hypothetical protein, QJT70586.1 | Hypothetical protein, AGI61421.1 | Hypothetical protein, QIN98213.1 | Hypothetical protein, QHR65330.1 | Hypothetical protein, QHR74769.1 | Hypothetical protein,QHR70486.1 |
| 46 | Hypothetical protein, QJT70587.1 | Hypothetical protein, AGI61423.1 | Hypothetical protein, QIN98214.1 | Hypothetical protein, QHR65331.1 | Hypothetical protein, QHR74770.1 | Hypothetical protein,QHR70482.1 |
| 47 | Putative polynucleotide 5' kinase/3' phosphatase, QJT70588.1 | Hypothetical protein, AGI61425.1 | Putative polynucleotide 5' kinase/3' phosphatase, QIN98216.1 | Putative kinase-phosphatase, QHR65333.1 | Putative polynucleotide 5' kinase/3' phosphatase, QHR74772.1 | Putative kinase-phosphatase, QHR70484.1 |
| 48 | Hypothetical protein, QJT70590.1 | Hypothetical protein, AGI61427.1 | Hypothetical protein, QIN98218.1 | Hypothetical protein, QHR65335.1 | Hypothetical protein, QHR74773.1 | Hypothetical protein, QHR70481.1 |
| 49 | Hypothetical protein,QJT70591.1 | Hypothetical protein,AGI61428.1 | Hypothetical protein,QIN98219.1 | Hypothetical protein,QHR65336.1 | Hypothetical protein,QHR74774.1 | Hypothetical protein,QHR70480.1 |
| 50 | Hypothetical protein,QJT70592.1 | Hypothetical protein,AGI61430.1 | Hypothetical protein,QIN98221.1 | Hypothetical protein,QHR65337.1 | Hypothetical protein,QHR74775.1 | Hypothetical protein,QHR70479.1 |
| 51 | Hypothetical protein, QJT70594.1 | Hypothetical protein, AGI61431.1 | Hypothetical protein, QIN98222.1 | Hypothetical protein, QHR65338.1 | Hypothetical protein, QHR74776.1 | Hypothetical protein,QHR70477.1 |
| 52 | Hypothetical protein, QJT70596.1 | Hypothetical protein, AGI61435.1 | Hypothetical protein, QIN98144.1 | Hypothetical protein, QHR65341.1 | Hypothetical protein, QHR74779.1 | Hypothetical protein,QHR70476.1 |
| 53 | Hypothetical protein, QJT70597.1 | Hypothetical protein, AGI61436.1 | Hypothetical protein, QIN98145.1 | Hypothetical protein, QHR65342.1 | Hypothetical protein, QHR74780.1 | Hypothetical protein,QHR70475.1 |
| 54 | Hypothetical protein, QJT70598.1 | Hypothetical protein, AGI61437.1 | Hypothetical protein, QIN98146.1 | Hypothetical protein, QHR65343.1 | Hypothetical protein, QHR74781.1 | Hypothetical protein,QHR70473.1 |
| 55 | Hypothetical protein, QJT70600.1 | Hypothetical protein, AGI61439.1 | Hypothetical protein, QIN98148.1 | Hypothetical protein, QHR65345.1 | Hypothetical protein, QHR74783.1 | Hypothetical protein,QHR70472.1 |
| 56 | Hypothetical protein, QJT70601.1 | Hypothetical protein, AGI61440.1 | Hypothetical protein, QIN98149.1 | Hypothetical protein,  QHR65346.1 | Hypothetical protein, QHR74784.1 | Hypothetical protein,QHR70471.1 |
| 57 | Hypothetical protein, QJT70603.1 | Hypothetical protein, AGI61442.1 | Hypothetical protein, QIN98150.1 | Hypothetical protein, QHR65347.1 | Hypothetical protein, QHR74786.1 | Hypothetical protein,QHR70470.1 |
| 58 | Terminase small subunit, QJT70604.1 | Hypothetical protein, AGI61443.1 | Terminase small subunit, QIN98151.1 | Terminase small subunit, QHR65348.1 | Terminase small subunit, QHR74787.1 | Terminase small subunit, QHR70469.1 |
| 59 | Terminase large subunit, QJT70605.1 | Hypothetical protein, AGI61444.1 | Terminase large subunit, QIN98152.1 | Terminase large subunit, QHR65349.1 | Terminase large subunit, QHR74788.1 | Terminase, large subunit, QHR70468.1 |

**Supplementary Table S5.** Detailed features of Maximum Parsimony trees generated using MEGA X in respect of individual marker genes.

| Feature | Protein | | | |
| --- | --- | --- | --- | --- |
|  | Terminase large subunit | Portal protein | ATP dependent helicase | DNA primase |
| Length of most parsimonious tree | 565 | 371 | 565 | 277 |
| Consistency index | 0.759259 | 0.818182 | 0.759259 | 0.724719 |
| Retention index | 0.911715 | 0.956306 | 0.911263 | 0.913732 |
| Composite index* | 0.827805  ( 0.692228) | 0.842890  ( 0.782432) | 0.827394  ( 0.691885) | 0.752097  (0.662199) |
| Total positions in final dataset | 521 | 422 | 521 | 218 |

* Values shown outside and in the parenthesis are for all sites and parsimony-informative sites,

respectively.

**Supplementary Table S6.** Detailed features of predicted conserved domains of 2019SD1.

| **ORF** | **Hit Type** | **PSSM-ID** | **Interval** | **E-Value** | **Accession** | **Short Name** |
| --- | --- | --- | --- | --- | --- | --- |
| *ORF*15 | superfamily | 227606 | 163- 755 | 4.91697e-16 | cl34971 | COG5281 superfamily |
| *ORF*31 | superfamily | 419756 | 241- 257 | 0.00369751 | cl21601 | zf-CHC2 superfamily |
| *ORF39* | superfamily | 398359 | 26- 58 | 0.00451549 | cl08422 | PLATZ superfamily |


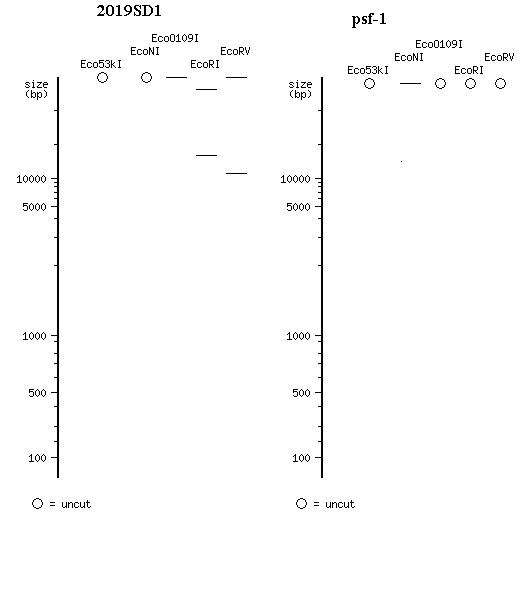


**Supplementary Figure S1.** Comparing cleavage site of *Shigella* virus 2019SD1with Psf-1

**Supplementary Figure S2.** Comparative analysis of various modules of 2019SD1 with related five phages using EasyFig program^39^. Proposed functional module clusters are indicated by the same colour. Boxed arrows depict the position and transcriptional direction of ORFs.
